# Single-cell Transcriptomic Architecture Unraveling the Complexity of Tumor Heterogeneity in Distal Cholangiocarcinoma

**DOI:** 10.1016/j.jcmgh.2022.02.014

**Published:** 2022-02-24

**Authors:** Hongguang Li, Lingxin Qu, Yongheng Yang, Haibin Zhang, Xuexin Li, Xiaolu Zhang

**Affiliations:** 1 Department of Hepatobiliary Surgery, Shandong Provincial Hospital, Cheeloo College of Medicine, Shandong University, Jinan, Shandong, China; 2 Department of Physiology and Pathophysiology, School of Basic Medical Sciences, Cheeloo College of Medicine, Shandong University, Jinan, Shandong, China; 3 Division of Genome Biology, Department of Medical Biochemistry and Biophysics, Karolinska Institutet, Stockholm, Sweden

**Keywords:** Copy Number Variation, Intra-tumor Heterogeneity, Single-cell RNA Sequencing, Tumor Microenvironment, CCA, cholangiocarcinoma, CNV, copy number variation, dCCA, distal cholangiocarcinoma, DEGs, differentially expressed genes, eCCA, extrahepatic cholangiocarcinoma, FDR, false discovery rate, iCCA, intrahepatic cholangiocarcinoma, IHC, immunohistochemistry, M, malignant, N, normal, PBS, phosphate-buffered saline, PCA, principal component analysis, qPCR, quantitative polymerase chain reaction, SCENIC, single-cell regulatory network inference and clustering, scRNA-seq, single cell RNA sequencing, TFFs, trefoil factors, TFs, transcription factors, TME, tumor microenvironment, UMAP, uniform manifold approximation and projection for dimension reduction, UMI, unique molecular identifier

## Abstract

**Background & Aims:**

Distal cholangiocarcinoma (dCCA) are a group of epithelial cell malignancies that occurs at the distal common bile duct, and account for approximately 40% of all cholangiocarcinoma cases. dCCA remains a highly lethal disease as it typically features remarkable cellular heterogeneity. A comprehensive exploration of cellular diversity and the tumor microenvironment is essential to depict the mechanisms driving dCCA progression.

**Methods:**

Single-cell RNA sequencing was used here to dissect the heterogeneity landscape and tumor microenvironment composition of human dCCAs. Seven human dCCAs and adjacent normal bile duct samples were included in the current study for single-cell RNA sequencing and subsequent validation approaches. Additionally, the results of the analyses were compared with bulk transcriptomic datasets from extrahepatic cholangiocarcinoma and single-cell RNA data from intrahepatic cholangiocarcinoma.

**Results:**

We sequenced a total of 49,717 single cells derived from human dCCAs and adjacent tissues, identifying 11 distinct cell types. Malignant cells displayed remarkable inter- and intra-tumor heterogeneity with 5 distinct subsets were defined in tumor samples. The malignant cells displayed variable degree of aneuploidy, which can be classified into low- and high-copy number variation groups based on either amplification or deletion of chr17q12 - chr17q21.2. Additionally, we identified 4 distinct T lymphocytes subsets, of which cytotoxic CD8+ T cells predominated as effectors in tumor tissues, whereas tumor infiltrating FOXP3+ CD4+ regulatory T cells exhibited highly immunosuppressive characteristics.

**Conclusion:**

Our single-cell transcriptomic dataset depicts the inter- and intra-tumor heterogeneity of human dCCAs at the expression level.

**Summary**

This work depicts the intra-tumor heterogeneity landscapes of distal cholangiocarcinoma at single-cell expression level.

Cholangiocarcinoma (CCA) arises from the epithelial lining of the biliary tree. Based on the anatomical locations, CCAs are commonly classified into intrahepatic cholangiocarcinoma (iCCA), perihilar cholangiocarcinoma, and distal cholangiocarcinoma (dCCA); the latter one refers to a subtype emerging in the area between the origin of the cystic duct and ampulla of Vater.^1^^,^^2^ dCCA remains a highly lethal disease due to its increasing diagnostic incidence and high mortality rates. When diagnosed, the majority of cases have already reached the advanced stage because of being asymptomatic or having nonspecific symptoms at an early stage.^3^ Surgical resection and subsequent adjuvant therapy can improve the overall survival rate for dCCA, but optimal adjuvant treatment strategy has not yet been established. The recurrence rate after surgical resection remains high, with a median overall survival for patients with dCCA after surgery ranging from 35 to 48 months.^4^, ^5^, ^6^ The prognosis is extremely poor for patients with unresectable tumor due to the lack of available treatment options.

Compared with the other subtypes of CCA, the molecular landscape of dCCA remains poorly understood as little progress has occurred for it. Several studies using large-scale bulk genomic and transcriptomic data revealed some critical gene mutations and aberrant signaling pathways in dCCA pathogenesis. *KRAS*, *TP53*, *ARID1A*, and *SMAD4* were the most prevalent mutations.^7^ CCA, including dCCA, is featured by profound genetic heterogeneity and rich tumor microenvironment (TME) comprising various cell types such as tumor cells, infiltrating immune cells, endothelial cells, and extracellular components. As bulk profiling limits the ability to capture tumor heterogeneity, deciphering the molecular profiles at the subclone or single-cell level is important for understanding the biology, the oncogenic landscape, and the complex interaction with TME in dCCA, which could lead to optimum therapies with improvement in patient survival.

Single-cell RNA sequencing (scRNA-seq) analysis represents as a powerful tool for illustrating cellular diversity and intercellular communication at single-cell resolution.^8^ It has been applied to multiple tumor studies to help dissect cellular components, identify subsets of given cell type, and explore cross-talks between tumor cells and stroma cells in the microenvironment.^9^, ^10^, ^11^ In such a way, it has strongly improved our knowledge of tumor pathogenesis and facilitated the screening of potential tumor biomarkers and promising therapy targets.

In the current study, we applied a droplet-based scRNA sequencing platform (10x Genomics) to profile single-cell transcriptomics of 4 human dCCA samples and 3 adjacent biliary tract tissue samples from 4 patients with dCCA. A single-cell transcriptome atlas was constructed, and a high level of inter and intra-tumor heterogeneity in dCCA samples was identified. Moreover, we explored the genomic alterations of malignant cells, identifying the underlying malignancy-driven mechanisms and confirming the scRNAseq data. By comparing with bulk transcriptomic datasets from extrahepatic cholangiocarcinoma (eCCA), including perihilar cholangiocarcinoma and dCCA samples, we demonstrated big differences in the findings between the 2 technologies and highlighted the revolutionary improvements made in biology research by single-cell approaches. Moreover, we compared our single-cell RNA data of dCCA with that of iCCA, confirming the concept that iCCA and dCCA were 2 different molecular entities.

## Results

### Single-cell Transcriptomic Analysis Revealed the Spectrum of Cell Populations in Human dCCAs

**Figure 1:**
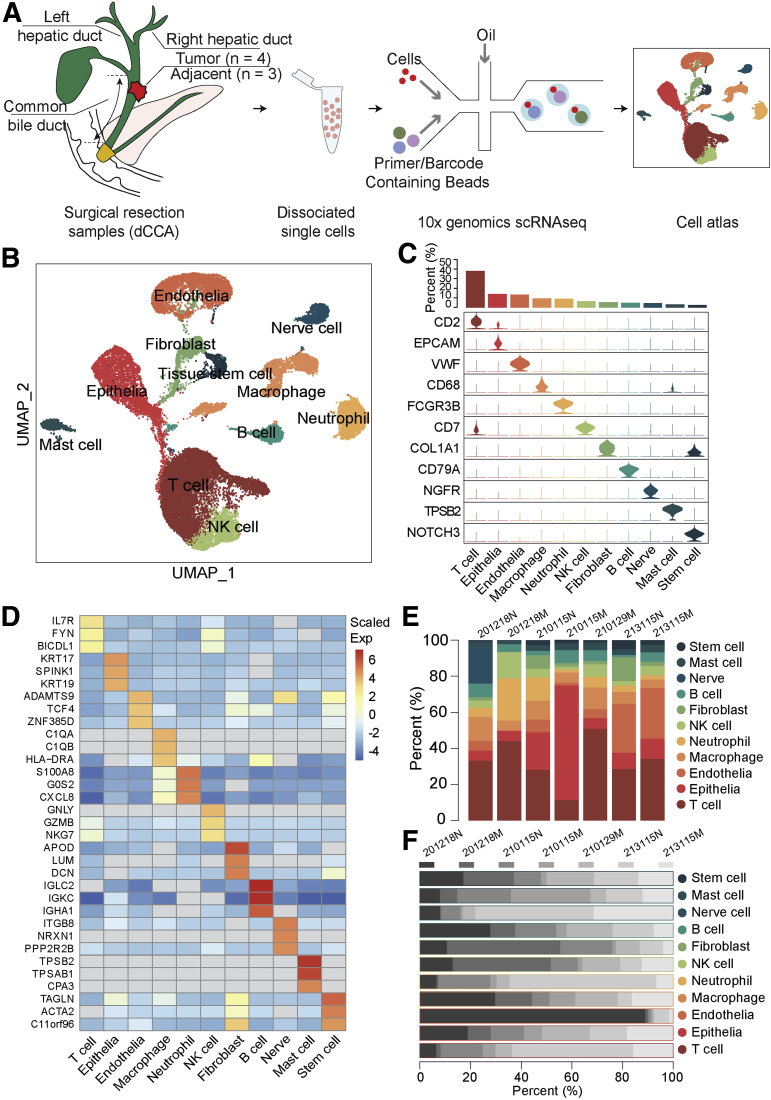
**A single-cell atlas of human dCCA.***A*, Schematic diagram of scRNA-seq analysis workflow. Human dCCAs and adjacent tissues are dissociated into single cells and sequenced using 10x Genomic platform. *B*, UMAP embedding of 30,860 cells from normal biliary duct (n = 3) and dCCA (n = 4) samples. Cells are colored by cell type. *C*, Violin plots showing marker genes and the percentage of each cell type. *D*, The top 3 DEGs for each cell type. *E*, Bar plot showing the proportion of cell types in each sample. *F*, Bar plot showing the proportion of each sample in each cell type.

**Figure 2:**
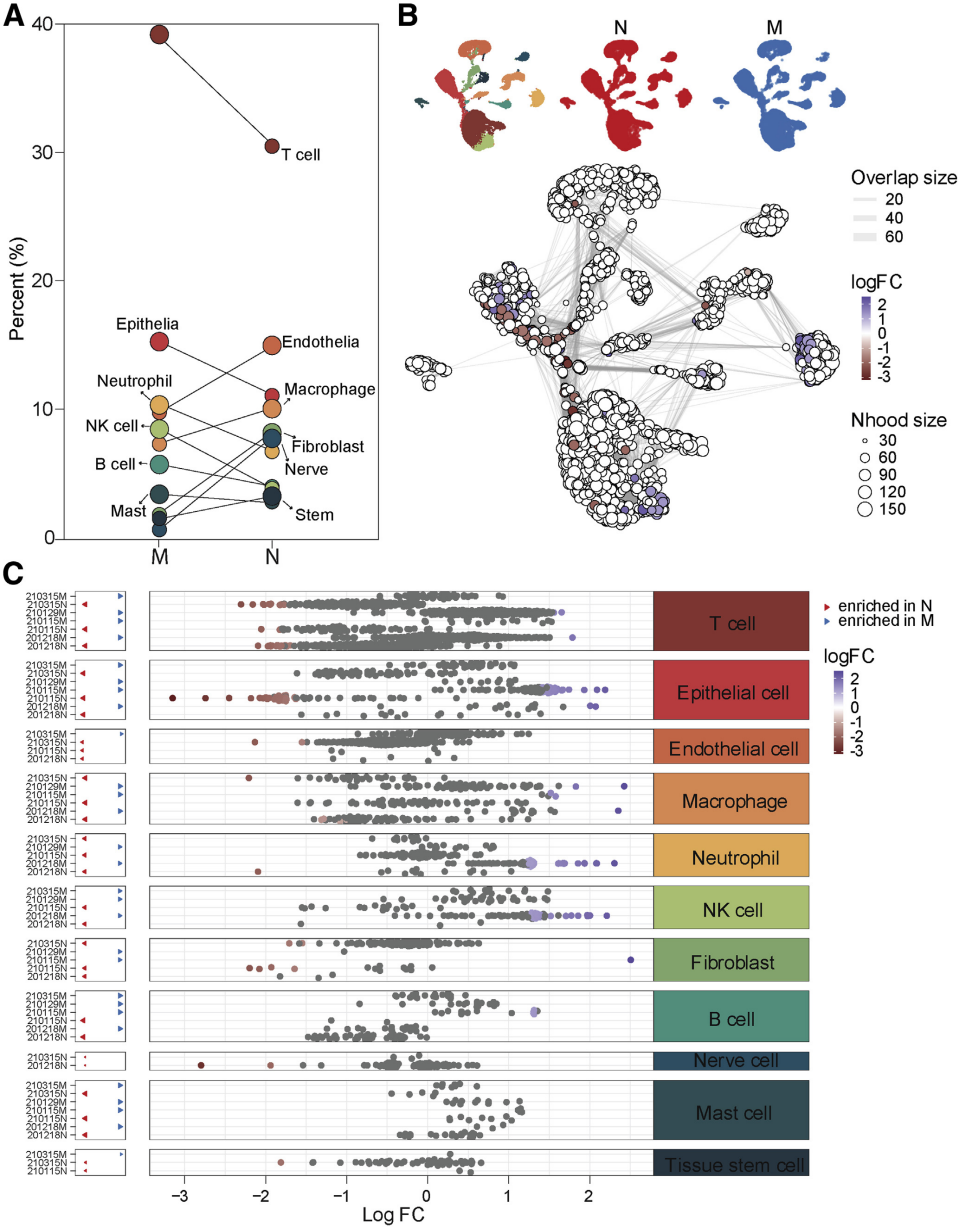
**Differential abundance between normal (N) and malignant (M) samples.***A*, Percentage shifts of all cell types between N and M. *B*, UMAP embedding of all cells colored by 2 conditions, *red* for N and *blue* for M (*upper panel*); graph representation of neighborhoods identified by Milo (*lower panel*). Nodes are neighborhoods, colored by their log fold change between M and N. Non-differential abundance neighborhoods (FDR = 25%) are colored *white*, and sizes correspond to the number of cells in a neighborhood. Graph edges depict the number of cells shared between adjacent neighborhoods. The layout of nodes is determined by the position of the neighborhood index cell in the UMAP embedding of single cells. *C*, Beeswarm plot showing the distribution of log fold change in abundance between M and N in neighborhoods from different cell type clusters. Differential abundance neighborhoods at FDR = 25% are colored.

To explore the diverse cellular components and tumor heterogeneity in human dCCA tissues, we applied 10x Genomics scRNA-seq and generated single-cell transcriptomic profiles of 4 treatment-naïve dCCA samples and 3 matched adjacent normal biliary duct tissues from 4 patients with dCCA (Figure 1, *A* [49,717 cells]). All patients underwent pancreaticoduodenectomy. For ethics reasons, we could not get any healthy or normal biliary duct tissue from other conditions as control; instead, we used matched adjacent normal biliary duct tissues in the current study. The normal biliary duct tissues were picked as far as the upper surgical margin. The pathological diagnosis was confirmed by clinical pathologists as well as the confirmation of the normal biliary duct. The detailed clinical characteristics of those patients are listed in Table 1. After stringent filtering, 30,860 cells were retained for further analysis using methods implemented in the Seurat software suite.^12^ Batch effects as well as variations in gender, age, and tumor stage among different patients were eliminated using Harmony^13^ tool to confirm that cells from multiple samples were mixed uniformly. After gene expression normalization, principal component analysis (PCA) and uniform manifold approximation and projection for dimension reduction (UMAP) were applied respectively for dimensionality reduction and clustering. All high-quality single cells were clustered and annotated into 11 distinct cell types with known canonical marker genes, including T cells (10,592 cells; 34.32%; with marker gene *CD2*), epithelial cells (3946 cells; 12.78%; marked with *EPCAM*), endothelial cells (3760 cells; 12.18%; marked with *VWF*), macrophages (2610 cells; 8.46%; marked with *CD68*), neutrophils (2508 cells; 8.13%; with marker gene *FCGR3B*), natural killer (NK) cells (1789 cells; 5.80%; marked with *CD7*), fibroblast cells (1481 cells; 4.80%; marked with *COL1A1*), B cells (1382 cells; 4.48%; marked with *CD79A*), nerve cells (1251 cells; 4.05%; with marker gene *NGFR*), mast cells (870 cells; 2.82%; with marker gene *TPSB2*), and tissue stem cells (671 cells; 2.17%; marked with *NOTCH3*) (Figure 1, *B and C*). Thus, we identified a broad range of cell types in human dCCA samples. The top differentially expressed genes (DEGs) for each cell type are shown in Figure 1, *D* and listed in Supplementary Table 1, confirming the precise annotation. The proportion of each cell type fluctuated greatly among samples (Figure 1, *E and F*), and we observed perturbations of all cell types between normal and malignant samples (Figure 2, *A*). We quantified shifts in abundance of cell types in response to dCCA malignancy in our study applying miloR tool,^14^^,^^15^ identifying 2446 neighborhoods spanning the KNN graph (*k* = 30), of which 77 showed evidence of differential abundance (false discovery rate [FDR] = 25%) (Figure 2, *B*) between normal (N) and malignant (M) conditions. Moreover, we compared differential abundance results with all discrete cell clusters identified previously, recovering differentially abundant neighborhoods in all clusters except the mast cell subset (Figure 2, *C*).

**Table 1:** Clinical Characteristics for Enrolled Patients

Patient ID	Gender	Age	Sample ID	Pathology	Tumor grade	TNM	SC count	Cell viability, %	nGene	nUMI
201218	Male	73	201218M	M	Moderately to poorly differentiated	T3N0M0, IIB	9636	87.93	1544	3959
201218	Male	73	201218N	N			7524	77.51	1848	4661
210115	Male	53	210115M	M	Moderately to poorly differentiated	T2N0M0, IIA	6825	89.71	4287	15,315
210115	Male	53	210115N	N			5947	88.96	2367	6124
210129	Male	60	210129M	M	Highly to moderately differentiated	T3N1M0, IIB	5369	90.61	2643	7769
210315	Female	58	210315M	M	Moderately differentiated	T3N0M0, IIB	6265	90.94	2505	7215
210315	Female	58	210315N	N			8151	82.09	2530	6587

*M*, Human dCCA tumor specimen; *N*, adjacent biliary duct tissue; *SC*, single cell; *UMI*, unique molecular identifier.

### Subsets of Malignant Cells Demonstrated Inter- and Intra-tumor Heterogeneity in Human dCCAs

**Figure 3:**
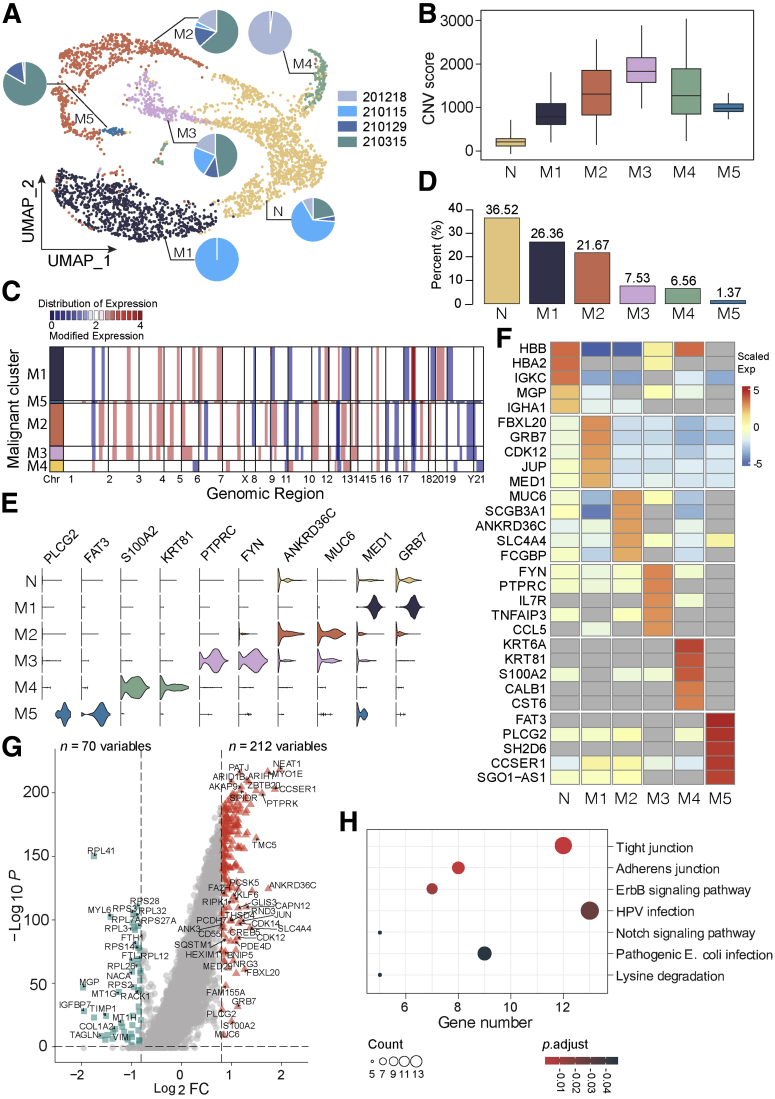
**Comprehensive cellular overview and heterogeneity of the malignant component in human dCCAs.***A*, UMAP plot of normal epithelial cells and five malignant subsets. Pie charts for each subset showing the contributing percentage of cells from each patient. *B*, Box plot showing the CNV signals for each epithelial subtype. *C*, Inferred CNV based on scRNA-seq data divided by malignant subtypes. The color bar (*blue, white, red*) represents the value of copy number from 0 to 4, respectively, and *red* means amplification, whereas *blue* indicates deletion. *D*, Bar plot showing the proportion of each epithelial cell subset. *E*, Violin plots showing marker genes of epithelial cell subgroup. *F*, Heat map showing the top DEGs in each epithelial cell subset. *G*, Volcano plot indicating the DEGs between all malignant epithelial cells and normal epithelial cells. *Red triangles* represent upregulated genes in malignant cells, whereas *green squares* indicate upregulated ones in normal cells. |Log2FC| ≥ 0.8; *P*-value ≤ .05. *H*, Kyoto Encyclopedia of Genes and Genomes analysis demonstrating the top signaling pathways in which those highly expressed genes in malignancy enriched.

We extracted and investigated all epithelial cells further and identified 6 main subclusters (Figure 3, *A*). As almost all cells in cluster N came from adjacent non-carcinoma tissues, we defined cluster N as normal epithelial cell subset and used it as reference for copy number variation (CNV) analysis. All the other 5 subsets (M1–M5) were defined as malignant, showing variable degree of CNV scores (Figure 3, *B and C*). Subcluster M1 was the most predominant malignant subset, whereas M5 was the minority malignant subcluster (Figure 3, *D*). The 5 malignant subtypes exhibited a great degree of inter and intra-tumor heterogeneity. Each tumor sample contained at least 3 different malignant cell subsets, whereas cells in each malignant subset originated from at least 2 tumor samples (Figure 3, *A*). The top DEGs of each malignant subtypes are shown in Figure 3, *E and F*. Subcluster M1 expressed a high level of epithelial-mesenchymal transition marker mediator subunit 1 (*MED1*) and growth factor receptor bound protein 7 *GRB7*.^16^^,^^17^ Subcluster M2 malignant cells were characterized by high expression levels of *ANKRD3BC* and *MUC6*, both of which were frequently mutated in cancer, and *MUC6* was linked to strong immune response. The top DEGs of M3 included *FYN* and *PTPRC*. M4 expressed a high level of S100A2 and cell differentiation-associated gene *KRT81*. S100 protein family members were commonly dysregulated in various tumors, and a high level of S100 proteins was linked with advanced tumor stage as well as worse prognosis.^18^^,^^19^ The last malignant subtype, M5, exhibited high expression levels of *FAT3* and *PLCG2*. Both genes have been reported to show recurrent mutations in cancer and are associated with a poor prognosis.^20^^,^^21^ Next, we compared the transcriptomic data between all malignant cells and normal epithelial cells, identifying 212 up-regulated genes in malignant and 70 relatively high-expressed genes in normal tissue (Figure 3, *G*). Kyoto Encyclopedia of Genes and Genomes analysis demonstrated that those highly expressed genes in malignancy were enriched in, for instance, cell junction, ErbB and Notch signaling pathways (Figure 3, *H*). To check whether the epithelia in malignant samples exhibit any abnormalities in comparison to normal, we investigated the transcriptomic landscapes of the 2 types of epithelia. In total, we identified 44 aberrantly up-regulated genes in epithelia of malignant samples, whereas 59 genes were relatively over-expressed in the epithelia of normal samples; the majority of both were ribosome genes. After investigation of the enriched signaling pathways, we found that the up-regulated genes in the epithelia of malignant samples were enriched in such ways as antigen processing and presentation, protein process, and interleukin-17 signaling pathways.^22^ This indicated that the epithelia in malignant samples was activated in the TME and involved in the immune system activation processes.

**Figure 4:**
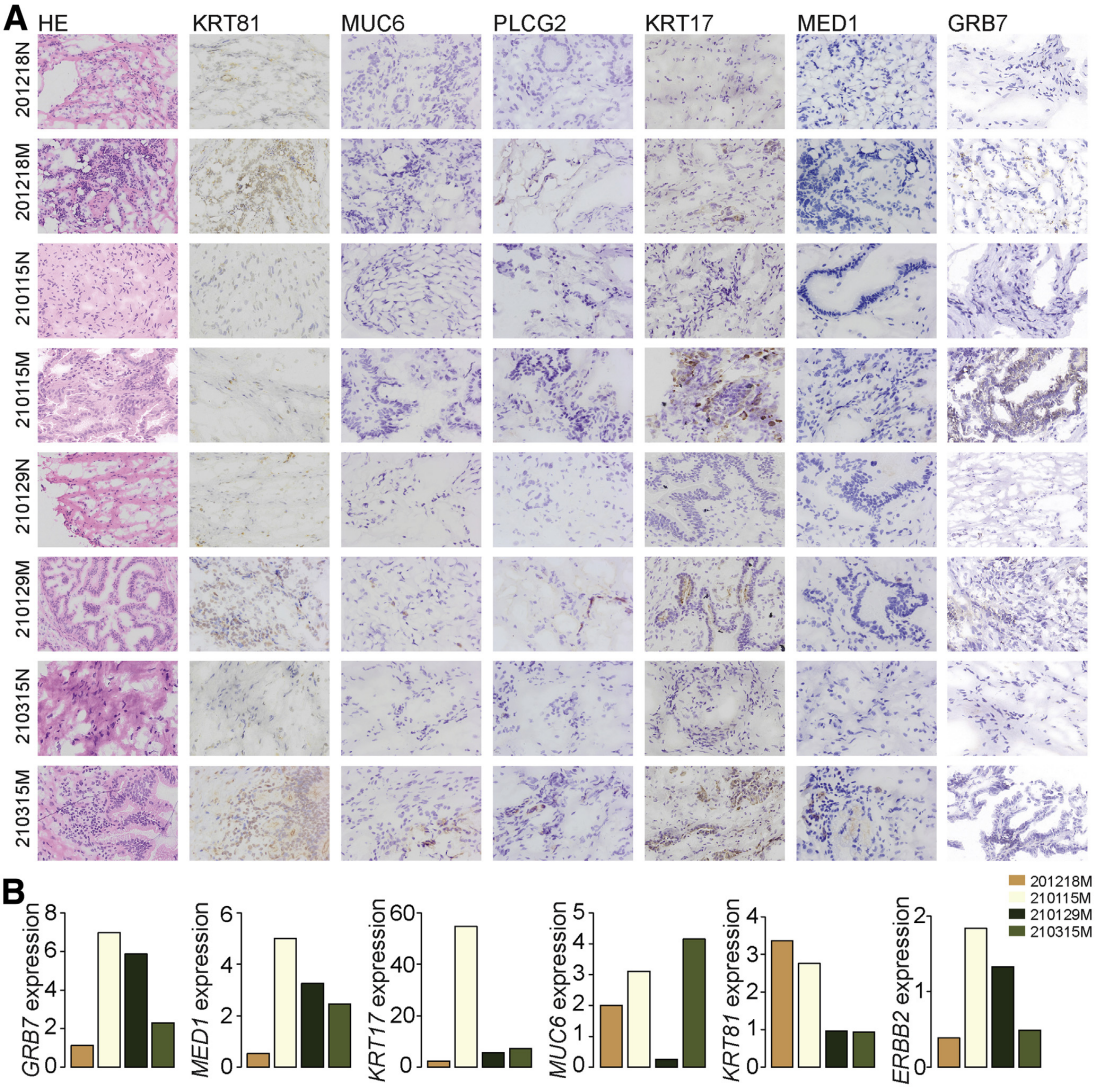
**Expression validation for marker genes.***A*, Hematoxylin and eosin and IHC staining showing the expression of selected marker genes in each sample and adjacent tissues. *B*, qPCR validation showing the fold change of expression for selected markers in each tumor sample compared with its matched adjacent normal sample.

The top DEGs of each subcluster were confirmed with immunohistochemistry (IHC) and quantitative polymerase chain reaction (qPCR) approaches, and the morphological overview of each sample was shown as well (Figure 4). The validated gene expression level was consistent with the single-cell transcriptome data, especially at the protein level (for instance, *GRB7* was demonstrated to be highly expressed in sample ‘210115’ [Figure 4, *A and B*] at both the protein and mRNA levels, detected by IHC and qPCR, respectively), which was the main sample origin of M1 (Figure 3, *A*). Correspondingly, *GRB7* was the top one DEG of M1 (Figure 3, *E and F*). Moreover, *MUC6* was exclusively detected positive in sample ‘210315’ by IHC (Figure 4, *A*), and it was the top DEG in M2 and M3 (Figure 3, *E and F*), both of which contained malignant cells mainly from sample ‘210315’ (Figure 3, *A*).

**Figure 5:**
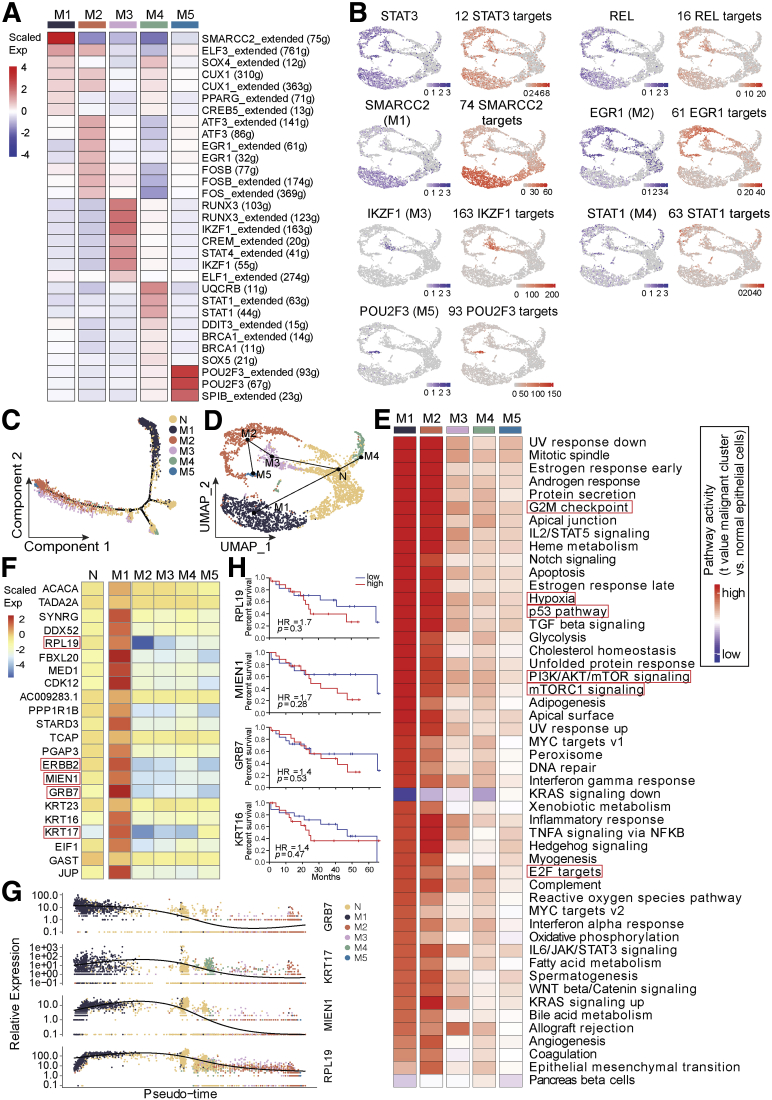
**Transcriptomic heterogeneity of malignant cells in human dCCA tissues.***A*, Heat map of the t-value for the area under the curve score of expression regulation by TFs, as estimated using SCENIC. *B*, Feature plots showing the distributions of active TFs and their targets in all malignant cells and in each malignant subgroup. UMAP plots of epithelial cells, color-coded for the expression of TFs (*purple*), for the area under the receiver operating characteristic curve of the estimated regulon activity of these TFs (*red*). *C* and *D*, Pseudo-time trajectory plots of all epithelial cell global transcriptomes by Monocle (*C*) and Slingshot (*D*). *E*, Differences in pathway activity (scored per cell by gene set variation analysis) in all malignant cell subclusters. *F*, Heat map showing expression level of all detected genes within chromosome region chr17q12 - chr17q21.2. *G*, The expression of selected genes (*GRB7*, *KRT17*, *MIEN1*, and *RPL19*) along the pseudo-time trajectory. *H*, Kaplan-Meier survival curve of selected gene expression using the median group cutoff showed the close relationship with patient overall survival probability in patients with CCA from The Cancer Genome Atlas data.

Moreover, we employed the single-cell regulatory network inference and clustering (SCENIC) method to explore all malignant epithelial cell subsets and identify the underlying transcription factors (TFs) in the different signatures (Figure 5, *A*). We found common underlying TFs for all malignant subsets, such as STAT3 and Rel (Figure 5, *B*); we also identified SMARCC2, EGR1, IKZF1, STAT1, and POU2F3 as the representative underlying TFs in M1 to M5, respectively (Figure 5, *A and B*). The expression of those TFs with their targets showed a consistent distribution manner (Figure 5, *B*). All of those TFs have been shown to play vital roles in the tumorigenesis and tumor progression processes.^23^, ^24^, ^25^, ^26^, ^27^ Interestingly, the top 2 DEG of M1- *GEB7*, was a strong target of SMARCC2; one of the targets of EGR1 was *MUC6*, which was the most highly expressed gene in M2; *FYN*, which was the top 1 DEG of M3, was among the targets of all representative TFs of M3 - IKZF1, RUNX3, CREM, and STAT4. Similarly, *FAT3* and *PLCG2*, both of which were the marker genes of M5, were targets of POU2F3 and SPIB, respectively.

Pseudo-time trajectory plot of the global transcriptomes for all epithelial cells showed that normal epithelial cells and each malignant cell subgroup formed a continuum, but malignant subgroups separated with each other, harboring distinct expression features, confirmed the heterogeneity of malignant subclones in human dCCAs (Figure 5, *C*). We also applied R package Slingshot^28^ to uncover the development trajectory of all epithelial cells and mapped the identified paths to UMAP projection for visualization. It demonstrated that the normal epithelial cell group formed as a root, giving 2 main trajectory branches separating M1 with M2, M3, and M5 (Figure 5, *D*). Gene set variation analysis was applied, indicating that all M subsets shared common activated signatures, such as PI3K/AKT/mTOR, mTORC1, p53, hypoxia, and the activation of cell cycle (G2M checkpoint, E2F targets) signaling pathways. M1 and M2 sub-groups shared the most common pathways (Figure 5, *E*).

### Malignant Cells Demonstrated Opposite Alteration Status of Chromosome 17

Interestingly, when we investigated the CNV data in detail, it was illustrated that all 5 malignant subsets could be classified roughly into 2 groups, the low-CNV group (M1) and the high-CNV group (M2-M5) (Figure 3, *B*), which was also separated into 2 branches on the pseudo-time trajectory plots (Figure 5, *C and D*). The low- and high-CNV M groups were characterized by either amplification or deletion of chr17q12 - chr17q21.2, respectively (Figure 3, *C*). This region involved multiple remarkable tumor-related genes (for instance *RPL19*, *ERBB2*, *MIEN1*, *GRB7*, *KRT17*, et al). All genes were highly expressed in M1, whereas they showed a relatively low expression level in M2 to M5 compared with the N group (Figure 5, *F*). The expression of selected genes (*RPL19*, *MIEN1*, *GRB7*, and *KRT17*) along the pseudo-time was defined as well, which showed a distinguishable expression distribution among subgroups (Figure 5, *G*). The Kaplan-Meier survival curve of most gene expression within this region using the median group cutoff showed the close relationship with patient overall survival probability in patients with CCAs from The Cancer Genome Atlas data (Figure 5, *H*). IHC staining and qPCR confirmed the expression alterations of selected genes (*KRT17* and *GRB7*) within this region for different subsets (Figure 4). Both *KRT17* and *GRB7* were highly expressed in sample ‘210115M’, which contained mainly M1 subgroup with genomic amplification of chr17q12.

### Cytotoxic CD8+ T Cells and Immunosuppressive Tumor-infiltrating Tregs Were Enriched in Human dCCA Tumors

**Figure 6:**
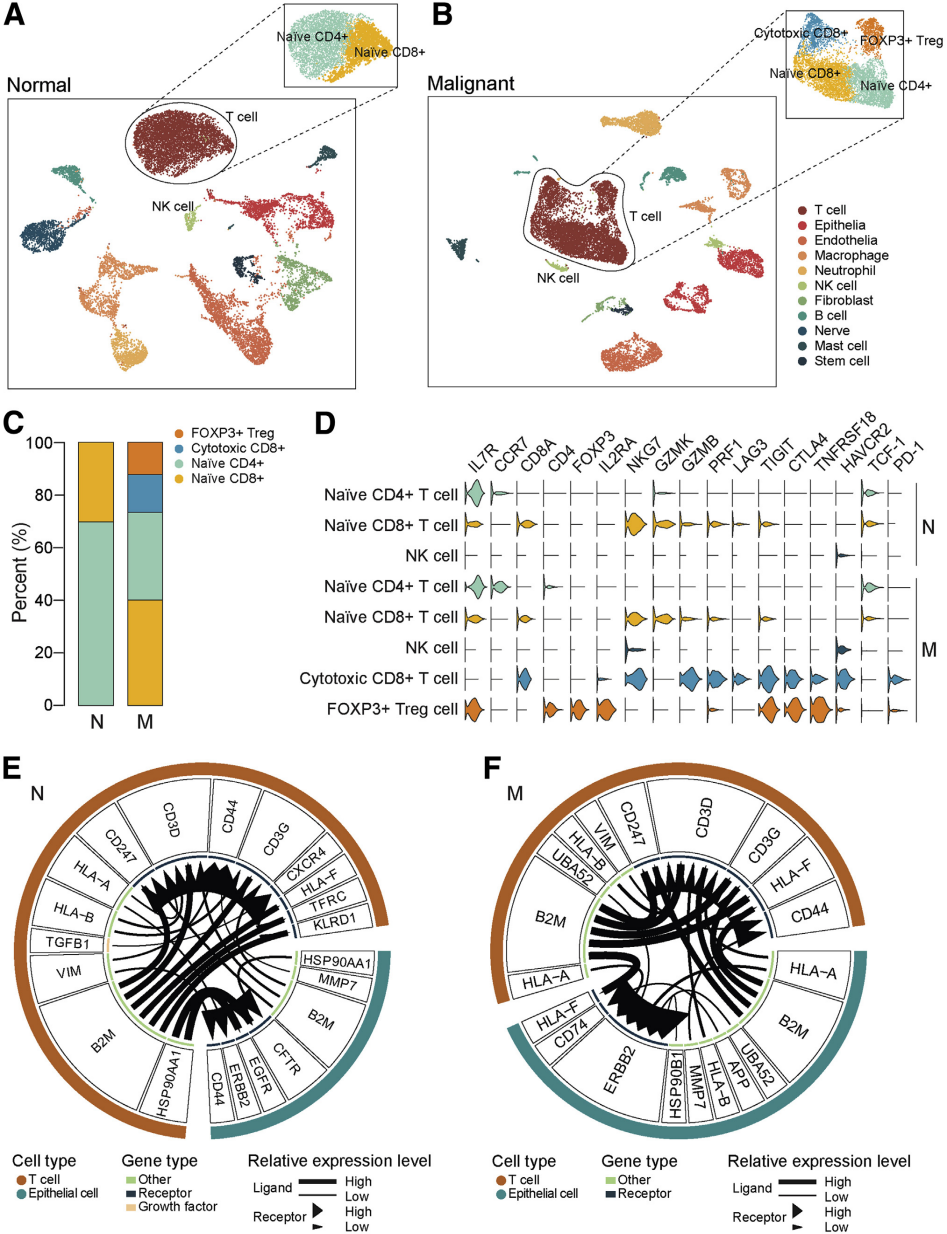
**Infiltrating immune cell subtypes landscape in human dCCAs.***A and B*, T cell subtypes identified in either normal (N) (*A*) or malignant (M) tissues (*B*). *C*, The proportion of each T cell subtype in N and M samples. *D*, Violin plots showing marker genes of each immune cell subgroup. *E and F*, Ligand-receptor interactions prediction network between T cells and epithelial cells in N (*E*) and M (*F*) samples. In the circus, the *lines* and *arrowheads* inside are scaled to indicate the correlations of the ligand and receptor. *P*-value < .05 is considered statistically different.

Tumor-infiltrating immune cells are highly heterogeneous and have been shown to play important roles in immunotherapy. In the current study, in the non-carcinoma biliary tract tissues, only naïve CD4+ and naïve CD8+ T cells were detected (Figure 6, *A*), whereas in cancer tissues, 4 distinct T cell subclusters were identified, as the emergence of cytotoxic CD8+ T cells and FOXP3+ Treg cells (Figure 6, *B*). The percentage of T cells in each cluster was shown in Figure 6, *C*, indicating naïve T cells were predominant in both tumor and non-tumor tissues. The cytotoxic CD8+ T cells were characterized by a high expression level of cytotoxic markers such as *GZMB* and perforin (*PRF1*), as well as a certain number of exhaustion markers, such as lymphocyte-activation gene 3 protein (*LAG3*), T cell immunoreceptor with Ig and ITIM domains (*TIGIT*), and T cell immunoglobulin mucin receptor 3 (*HAVCR2*), suggesting those cytotoxic CD8+ T cells became exhausted. The FOXP3+ Treg cells showed prominent expression levels of immunosuppression markers such as *TIGIT*, cytotoxic T lymphocyte antigen 4 (*CTLA4*), and TNFR-related protein (*TNFRSF18*). The effector T cells expressed a moderate level of programmed cell death-1 (*PD-1*). The NK cell clusters from either non-cancerous tissues or cancer tissues exhibited no significant individual features, and did not show any signs of activation, meaning the cytotoxic CD8+ T cells were the main effectors in dCCAs (Figure 6, *D*).

Next, we investigated the intercellular communication landscapes between T cells and epithelial cells in either normal tissue or tumor tissue using iTALK,^29^ which indicated that ERBB2 receptor was enhanced in tumor tissues during intercellular communications between T cell and epithelial cells, suggesting that blocking the ERBB2 signaling may affect the proliferation effects in malignant cells (Figure 6, *E*).

### Single-cell Data Compared With Bulk Expression Profiles

**Figure 7:**
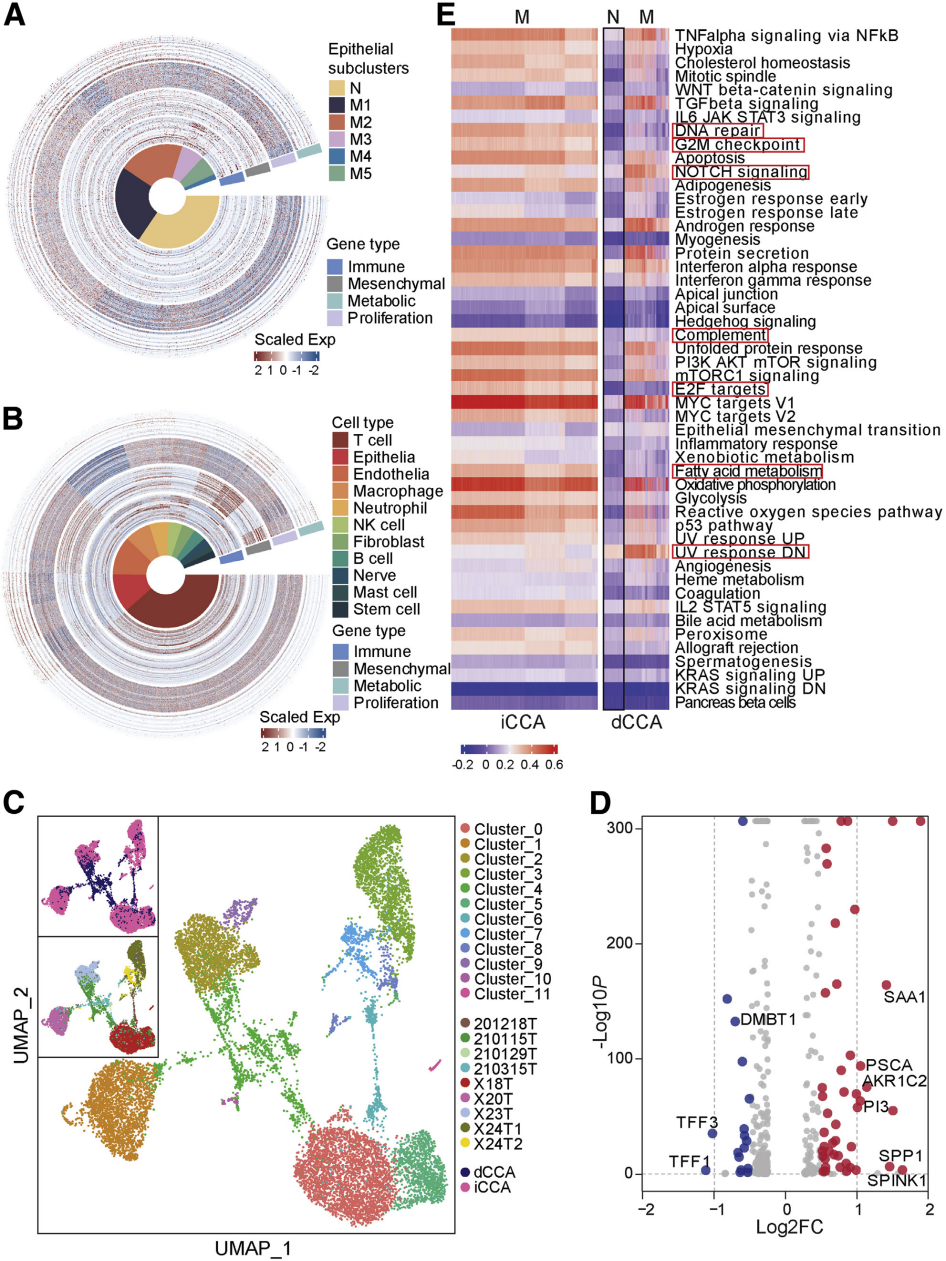
**Single-cell data comparison with bulk data and between dCCA and iCCA.***A and B*, Overlapping with a 174-gene classifier from bulk data by either single epithelial cell data (*A*) or all single-cell data (*B*). *C*, UMAP embedding of 12 cell subtypes of all malignant cells either from iCCA or dCCA. *D*, Volcano plot indicating the DEGs between iCCA and dCCA malignant cells. *Red* represents upregulated genes in iCCA, whereas *blue* indicates upregulated genes in dCCA. |Log2FC| ≥ 1; *P*-value < .05. *E*, Differences in pathway activity (scored per cell by Gene Set Enrichment Analysis) between iCCA and dCCA.

A recent published study analyzed the whole-genome expression profiles for 189 eCCA cases from an international multicenter cohort and classified all cases into 4 transcriptome-based molecular classes: Metabolic, Proliferation, Mesenchymal, and Immune, with each class showing distinct expression characteristics. The Metabolic class was dominated by gene expression data suggestive of deregulated metabolism of bile acids, fatty acids, and xenobiotics, showing a hepatocyte-like phenotype; the Proliferation class overexpressed MYC targets and featured activation of cell cycle signaling (E2F, mitotic spindle, and G2M checkpoint) and DNA repair pathways; the Mesenchymal class was enriched by genomic signals of epithelial-mesenchymal transition; and the Immune class was defined by upregulation of adaptive immune response genes. Moreover, a 174-gene classifier (genes are listed in Supplementary Table 2) was designed, composed of a maximum of 50 genes defining each class for externally validating the molecular classification of eCCA.^30^ Although this classification system was acquired from bulk tumors, the expression programs of individual cellular components should enable us to extract additional insights. We would define whether the molecular subtypes from bulk data could reflect differences in malignant programs and TME composition in single-cell data. First, we tried to overlap our single epithelial cell subsets with the molecular subtypes using the 174-gene classifier. Strikingly, the findings showed all malignant epithelial subsets spread mainly in the Proliferation group, without any big variations among subsets (Figure 7, *A*). It was consistent with gene set variation analysis findings, which showed all malignant subsets shared features of activation of G2M checkpoint and E2F targets pathways. However, when we expanded our analysis to include all TME components and classified again, the heat map showed that immune cells (T and B cells) fell in the subtype of Immune; those mesenchymal cells, like endothelial cell, fibroblasts, nerve cells, and tissue stem cells, were more prone to be classified as Mesenchymal; almost all cells except the nerve and neutrophil cells showed features of Proliferation, and the epithelial cells slightly exhibited features of Metabolic compared with other groups (Figure 7, *B*). Those findings raised the possibility that the Mesenchymal subtype in bulk data reflects high stromal representation and the Immune subtype is a reflection of immune cells infiltrated, rather than a distinct malignant cell program. A new set of genes for classifying subtypes should be defined in the case of the isolated epithelial malignant cell group in single-cell data.

### Single-cell Data Comparison Between dCCA and iCCA

CCA subtypes differ not only in their location but also in their etiopathogenesis and molecular aberrations, along with the emerging evidence pointing towards a different proposed cell of origin,^31^ as iCCA is mostly derived from trans-differentiation of adult hepatocytes or their progenitor cells, whereas eCCA is derived from ductal cells, suggesting that eCCA and iCCA are distinct molecular entities. To infer the molecular differences at the single-cell level, we compared our single-cell data of dCCA with a previous published work conducted on iCCA with scRNA-seq, with data downloaded from GSE138709.^11^ We extracted all malignant cells from the 2 datasets (11,186 cells) and eliminated the batch effects with the Harmony tool. In total, 12 subclusters were identified. Cells from iCCA or dCCA separated with no remarkable overlaps, which confirmed the different expression alteration landscapes between iCCA and dCCA (Figure 7, *C*). Interestingly, iCCA showed more significant interpatient heterogeneity than dCCA, with malignant cells from 4 patients with iCCA clustered independently, but it needed further study on larger cohort of patients for a validated conclusion. The DEGs between the 2 groups were shown in Figure 7, *D*, among which serine protease inhibitor Kazal type 1 (*SPINK1*) and phosphoprotein 1 (*SPP1*) were overexpressed in iCCAs, whereas trefoil factors (TFFs) (*TFF1*/*TFF3*) were overexpressed in dCCAs. *SPINK1* has been detected in multiple types of cancers including bladder, renal, prostate, and liver cancers.^32^ High levels of *SPINK1* presented as prognostic and diagnostic biomarkers in hepatocellular carcinoma, promoting cell proliferation and metastasis.^33^ Increased expression of *SPP1* promoted invasion and metastasis in various malignant tumors.^34^ TFFs function normally as secretory peptides to protect the gastrointestinal tract against mucosal damage.^35^ In pathology, TFFs played pivotal roles in oncogenic transformation, growth, and metastasis of tumors.^36^ In total, 45 genes were detected to be significantly upregulated in iCCA, whereas 17 were overexpressed in dCCA. Gene set enrichment analysis demonstrated that the 2 entities enriched in different activated signaling pathways. By comparison, iCCA was activated in DNA repair, G2M checkpoint, E2F, complement, and fatty acid metabolism pathways, whereas dCCA was enriched in NOTCH and UV response signaling pathways (Figure 7, *E*).

## Discussion

dCCA is an aggressive malignancy with poor prognosis and outcomes. As subtype of CCA, dCCA differs remarkably with iCCA but often resembles adenocarcinoma of the pancreatic head and represents a distinct molecular entity.^37^ In the past decade, significant efforts have been conducted to elucidate the molecular pathogenesis of CCA, but there is still limited understanding of the molecular landscape of dCCA, and no targeted therapy with clinical efficacy has been approved. Understanding the tumorigenesis and underlying molecular basis of dCCA is an unmet need. A comprehensive exploration of the transcriptomic profiles at the single-cell level can improve knowledge of dCCA pathogenesis and help in development of optimal therapy strategies. In the current study, we applied scRNA-seq to comprehensively delineate the transcriptomic landscape of human dCCA and elucidated the heterogeneity as well as the complex microenvironment in dCCA.

With the scRNA-seq approach, we annotated diverse distinct cell types in dCCAs, with the majority being T cells, which accounts for more than 30% of all cells; following is epithelial cells, including both normal and malignant epithelial cells. The percentage of each cell type in individual sample varies greatly, showing tremendous intertumor heterogeneity. ScRNA-seq analysis has been used to explore constituent malignant cell types in multiple cancer types, including gastric cancer,^38^ renal cell cancer,^39^ and others. In our study, scRNA-seq helped to define 5 different malignant subtypes, with each characterized by specifically DEGs, underlying TFs, cell trajectory, and copy number alterations. The tumor specimen of each patient contained at least 3 different malignant cell subsets, exhibiting a highly intratumor heterogeneity of tumor clones. Copy number differences are common aberrations. We identified that all 5 malignant subsets could be classified roughly into the low-CNV group and the high-CNV group, characterized by either amplification or deletion of chr17q12 - chr17q21.2, respectively. In a genomic profiling study with biliary tract cancers which included 101 dCCA samples, researchers identified that a 350-kb region at 17q12 was amplified in 32.6% samples, containing known oncogene *TBC1D3* and cytokine genes *CCL3L3* and *CCL4L2*. Event of amplification of 17q12 showed disease-free survival hazard ratio of 1.36 and overall survival hazard ratio of 1.46.^7^ In addition, 17q copy number gain has been illustrated to be a unique event prevalent in tumor cells of stomach origin and is only present in tumor cells from the short-term survivors.^40^ A star gene among the upregulated genes within this region was GRB2, with a number of compounds being screened as active.^41^ The clinical significance and the underlying genomics-driven powers for cellular diversity of the copy number alteration status of this region requires further validation strategy in the future with a large cohort of cases.

Cancer is a complex disease involving interactions between the tumor and the immune system.^42^ Studies showed Th1 cell and cytotoxic immune infiltration in human tumors was associated with a favorable clinical outcome, whereas a low density of T cells was associated with a poor prognosis.^43^ In the case of dCCA, the tumor immune microenvironment is less complicated than other tumor types, such as head and neck cancer,^44^ gastric cancer,^38^ and others, as we only defined cytotoxic CD8+ T cells as effector T cells as well as FOXP3+ Treg cells as immune tolerance cells. On the other hand, it has been known for a long time that more-differentiated effector T cells were less effective for in vivo antitumor properties compared with naïve and early effector T cells, and less-differentiated T cells are more therapeutically effective upon adoptive transfer.^45^ In our study, we found that the cytotoxic CD8+ T cells expressed a high level of exhaustion markers, like *LAG3*, *TIGIT*, and *HAVCR2*, suggesting those cytotoxic CD8+ T cells became exhausted. But the high level of naïve T cells in the microenvironment of dCCA may show promise as a potential immune therapy target.

By comparing our single-cell data with bulk transcriptome data of eCCA, we found that the malignant cells mainly fell into the Proliferation group, featured by the activation of cell cycle (E2F, mitotic spindle, and G2M checkpoint signaling), mTOR, and ERBB2. Those data suggested that anti-proliferative agents such as casein kinase II inhibitors^46^ and CDK4/6 inhibitors may imply potential therapeutic property for dCCA.

Subtypes of CCA arise through different extrinsic and intrinsic carcinogenic processes.^47^^,^^48^ Molecular landscapes differ significantly depending on the anatomic locations of CCA subtype (for example, *FGFR2* fusions are almost exclusively detected in iCCAs, whereas *PRKCA-PRKCB* fusions are found in eCCAs).^49^ Our observations confirmed iCCA and dCCA as 2 different molecular entities, by showing malignant cells from the 2 entities formed different clusters and expressed different DEGs, which enriched in distinguishable activated signaling pathways. Those findings exhibit importance not only for pathogenesis mechanism understanding but also for clinical decision-making purposes.

Taken together, our single-cell dataset provides a comprehensive transcriptomic landscape of human dCCA, revealing a high level of inter- and intra-tumor heterogeneity and unraveling key biological traits with potential clinical implications for dCCA. Our study supports the concept that the molecular scenario of dCCA is intrinsically different than iCCA, pointing out unique precision therapeutic approaches that can be implemented in clinical situations.

## Methods

### Human dCCA Samples

Human dCCA samples and paired adjacent normal biliary duct tissues were collected from the Department of Hepatobiliary Surgery of Shandong Provincial Hospital (Jinan, China). The enrolled patients with dCCA were newly diagnosed and treatment-naïve before undergoing surgical resection. None of the patients had autoimmune disorders or history of prior cancers. In total, 4 dCCA samples and 3 matched adjacent biliary duct tissues from 4 patients with dCCA were used in the study for single-cell transcriptomics analysis. The study was approved by the local ethics committee, and written informed consent was obtained from all patients. All authors had access to the study data and had reviewed and approved the final manuscript.

### Fresh Tissue Preparation and Single-cell Isolation

All the freshly resected surgical specimens were immediately washed with phosphate-buffered saline (PBS) and divided into 2 equal parts. One part was used for single-cell isolation and subsequent scRNA-seq library preparation, whereas the other part was stored at −80 °C for pathology examination and other validation experiments. Tissue digestion was incubated in a 15-mL tube containing 10 mL pre-warmed RPMI 1640 (ThermoFisher Scientific), 2 mg/mL dispase (Roche), 1 mg/mL type IV collagenase (Sigma), and 10 U/μL DNase I (Roche) for 60 minutes at 37 °C, then deactivated with 10% fetal bovine serum. Cell suspensions were filtered using a 70-μm filter and then centrifuged at 500 rpm for 6 minutes at 4 °C to pellet dead cells and red blood cells. The cells were washed twice and suspended in PBS with 0.5% bovine serum albumin (Sigma). Then, fluorescence-activated cell sorting system was used to load cell for detection of cell viability and cell concentration. Samples could be processed further with cell viability higher than 70%. We diluted cell concentration to 300 to 600 cell/μL for library preparation.

### Library Preparation and Sequencing

Viable cells were loaded into a well of a microfluidic chip to generate cDNA library using 10x Genomics Chromium Single Cell Gene Expression Solution platform (10x Genomics, Pleasanton, CA). Single-cell transcriptomic amplification and library preparation were performed by CapitalBio Technology Corporation (Beijing, China) using single-cell 3’ v3 (10x Genomics) according to the manufacturer’s instructions. Libraries were sequenced on Illumina NovaSeq6000 system (Illumina, Inc, San Diego, CA).

### Single-cell Data Processing and Cell Subsets Annotation

Raw data was processed with CellRanger (10x Genomics) and Seurat R package (version 4.0.5).^50^ Cell-barcode and unique molecular identifier (UMI) were extracted first, then RNA sequences were aligned to the reference genome (GRCh38), and reads mapped confidently to genome were used for subsequent analysis. Low-quality cells were removed according to the following criteria: cells had expressed gene counts fewer than 200 or more than 5000, or over 15% of UMIs derived from the mitochondrial genome. Additionally, all genes that were not detected in at least 3 single cells were discarded. All remaining cells were considered as high-quality cells, of which the gene expression matrices were normalized to the total cellular UMI counts. High variable genes were calculated using Seurat FindVariableGenes function with default parameters, and the top 2000 most variable genes were selected as representative for all genes for following PCA analysis and dimension reduction. Batch effects as well as variations in gender, age, and tumor stage among different samples were eliminated using the Harmony tool. Based on the elbow plot in which principal components were plotted as a function of the variability they accounted for and the heat maps of leading genes in each principal component, the top 15 significant harmonys were selected manually to perform dimension reduction; clusters were identified using FindClusters function (dims.use = 1:15, resolution = 0.5). The UMAP analyses were used for cluster visualization.^51^ Cell subsets (Seurat clusters) were annotated to known biological cell types using canonical marker genes.

### Differential Abundance Analysis With miloR

We applied miloR package^14^ to dissect differential abundance for our scRNAseq data. Briefly, milo uses a KNN graph computed based on similarities in gene expression space as a representation of the phenotypic manifold on which cells lie (*k* = 30, d = 15). A representative subset of neighborhoods was defined that span the whole KNN graph. For each neighborhood, we counted the number of cells from each sample and tested differential abundance in neighborhoods, while setting spatial FDR as 25%.

### Distinguishing Malignant and Nonmalignant Epithelial Cells

All epithelial cells were extracted for further analysis. Subclusters of epithelial cells were identified using FindClusters function after PCA analysis and dimension reduction as mentioned above. Batch effects among different samples were eliminated with the Harmony tool. Malignant epithelial cells were determined based on inferred CNVs, setting the subcluster of normal epithelial cells originated mainly from noncancerous tissue as reference. Initial CNVs for each region were estimated by inferCNV R package.^52^ The CNV score of each cell was calculated as quadratic sum of CNV region.

### SCENIC Analysis

After subsets of epithelial cells were defined, we employed the SCENIC package^53^ (version 1.2.4) to analyze the enriched transcriptome factors for each subtype. SCENIC reconstructed regulons and assessed the activity of these discovered regulons in individual cells. Specific regulons (ie, transcription factors and their target genes) for each epithelial subset were identified.

### Pseudo-time Analysis

Single epithelial cell trajectory analysis was performed using Monocle R package^54^ and Slingshot R package. For Monocle, first, a Cell DataSet matrix was created for single epithelial cells using the default parameters. Next, we used the marker genes of each cluster to define the progression of cell transition. Then, we entailed dimensionality reduction and trajectory construction with the ordering genes. The expression of selected genes along the pseudo-time was defined as well. For Slingshot, first, a minimum spanning tree was constructed for defining a global lineage structure. Principal curve was fitted onto the reduced dimension dataset to compute pseudo-time scores for each lineage predicting cell-level transcriptional states. UMAP projection was mapped with the identified paths to for visualization.

### IHC Analysis

Frozen tissue sections (4–6 μm) were fixed in 2-propanone for 10 to 20 minutes, then washed with PBS for 3 minutes 3 times. Endogenous peroxidase activity was quenched for 30 minutes in 10% hydrogen peroxide. To examine the expression pattern of candidate antibodies in dCCAs and adjacent tissues, sections were immunostained with primary antibodies overnight at 4 °C. The following antibodies were used in the current project: rabbit-anti-KRT17, rabbit-anti-PLCG2 (ABclonal, Wuhan, China), rabbit-anti-KRT81 (Servicebio, Wuhan, China), mouse-anti-MUC6, rabbit-anti-GRB7 (Abcam, Cambridge, UK) and rabbit-anti-MED1 (ThermoFisher, MA). The secondary antibody used for immunostaining was biotin-conjugated anti-rabbit or anti-mouse immunoglobulin (ZSGB-Bio, Beijing, China). The signal was detected using an ABC kit (ZSGB-Bio, Beijing, China), following the protocol of the manufacturer. Hematoxlin was used for counterstaining.

### Quantitative Reverse-transcription PCR

Total RNA was extracted using Trizol (ThermoFisher, Waltham, MA). EasyScript First-Strand cDNA Synthesis SuperMix was used for reverse transcription. The PCR mixture was prepared using SYBR Green qPCR SuperMix (Vazyme Biotech Co, Ltd, Nanjing, China). PCR was performed using an ABI PRISM 7500 Sequence Detection System (Foster City, CA). The primer sequences used for gene detection are listed in Table 2. All primers were designed using Primer Premier 5.0 (PREMIER Biosoft International, Palo Alto, CA). GAPDH was used as an internal expression control.

**Table 2:** qPCR Primers Used in the Current Study

Gene	Forward primer	Reverse primer
GAPDH	CAGGAGGCATTGCTGATGAT	GAAGGCTGGGGCTCATTT
GRB7	TGCAGTACGTGGCAGATGTG	GAAGATCCGAAGCCCCTTGT
MED1	CTGGAACGGCTCCATGCAA	CTTCTCCATGACTTGACGCAC
KRT17	CTCCTCCCAGAGGAAGAACTGG	TCTTGAGTCCTCTCTGCGTG
MUC6	TGGTGAACTCGTGGAAGGA	TGGCAGGTGGCAAAGGT
KRT81	AGGCTATGTGAAGGCATTGG	AAGTGGGGGATCACACAGAG
ERBB2	ACCCGCTGAACAATACCA	GGATCAAGACCCCTCCTT

*qPCR*, Quantitative polymerase chain reaction.

### Data Transparency

All the sequencing data related to the clinical samples described in this study have been deposited in the National Center for Biotechnology Information Sequence Read Archive with the following SRA accession: SUB11007007. All other datasets used and/or analyzed during the current study are available within the manuscript and its supplementary information files.

## Figures and Tables

**Supplementary Table 1:** The Top 30 Differentially Expressed Genes of Each Cell Type in the dCCA Ecosystem

Gene	p_val	avg_log2FC	pct.1	pct.2	p_val_adj	Cell type
IL7R	0	2.857819907	0.758	0.095	0	T cell
FYN	0	2.248950569	0.962	0.419	0	T cell
BICDL1	0	2.240326143	0.729	0.115	0	T cell
FAAH2	0	2.116057013	0.334	0.087	0	T cell
CAMK4	0	2.074403984	0.668	0.086	0	T cell
STAT4	0	1.990967921	0.845	0.19	0	T cell
BCL11B	0	1.982332052	0.754	0.108	0	T cell
PPP2R5C	0	1.967799479	0.903	0.416	0	T cell
ICOS	0	1.938624088	0.55	0.066	0	T cell
CD96	0	1.914143167	0.762	0.113	0	T cell
PDE3B	0	1.87273318	0.715	0.196	0	T cell
SKAP1	0	1.872352468	0.774	0.156	0	T cell
TRBC2	0	1.852902218	0.708	0.12	0	T cell
CNOT6L	0	1.826390391	0.866	0.382	0	T cell
PTPRC	0	1.813943528	0.973	0.404	0	T cell
CD2	0	1.786055532	0.746	0.098	0	T cell
PBX4	0	1.753179348	0.602	0.088	0	T cell
GZMK	0	1.715931909	0.347	0.034	0	T cell
CD247	0	1.694833662	0.75	0.109	0	T cell
CD3D	0	1.693257118	0.746	0.084	0	T cell
ITK	0	1.676364898	0.598	0.089	0	T cell
TNFAIP3	0	1.676018238	0.876	0.443	0	T cell
PPP1R16B	0	1.66280953	0.769	0.215	0	T cell
RHOH	0	1.66278223	0.802	0.234	0	T cell
CDC42SE2	0	1.660072066	0.942	0.502	0	T cell
TRAC	0	1.642384535	0.646	0.097	0	T cell
THEMIS	0	1.622233359	0.501	0.07	0	T cell
IL32	0	1.610984954	0.78	0.272	0	T cell
CD69	0	1.595429994	0.817	0.253	0	T cell
TRBC1	0	1.581554567	0.45	0.075	0	T cell
KRT17	0	4.534853267	0.453	0.049	0	Epithelial cell
SPINK1	0	4.163811343	0.596	0.061	0	Epithelial cell
KRT19	0	4.080768781	0.825	0.141	0	Epithelial cell
LCN2	0	3.959546363	0.666	0.083	0	Epithelial cell
SCGB3A1	8.06E-128	3.877080534	0.254	0.131	2.60E-123	Epithelial cell
MT1G	0	3.847091868	0.546	0.103	0	Epithelial cell
ERBB2	0	3.608416345	0.563	0.087	0	Epithelial cell
GRB7	0	3.550084194	0.505	0.035	0	Epithelial cell
TFF1	0	3.499223304	0.506	0.037	0	Epithelial cell
TFF3	0	3.407592422	0.545	0.062	0	Epithelial cell
KRT8	0	3.366359295	0.785	0.101	0	Epithelial cell
JUP	0	3.358535225	0.608	0.087	0	Epithelial cell
KRT18	0	3.226750562	0.774	0.112	0	Epithelial cell
PPP1R1B	0	3.18786528	0.508	0.043	0	Epithelial cell
OLFM4	0	3.077164181	0.426	0.041	0	Epithelial cell
ANXA4	0	3.04477107	0.758	0.22	0	Epithelial cell
MT1H	0	3.023401253	0.391	0.042	0	Epithelial cell
MIEN1	0	2.980164106	0.503	0.2	0	Epithelial cell
ELF3	0	2.957400736	0.678	0.06	0	Epithelial cell
CLDN4	0	2.951918348	0.653	0.07	0	Epithelial cell
RPL19	0	2.938058416	0.93	0.893	0	Epithelial cell
GPX2	0	2.937746247	0.614	0.055	0	Epithelial cell
MMP7	0	2.936717406	0.493	0.047	0	Epithelial cell
AGR2	0	2.911912742	0.628	0.059	0	Epithelial cell
LGALS4	0	2.845893248	0.658	0.051	0	Epithelial cell
S100A6	0	2.808406823	0.906	0.761	0	Epithelial cell
PIGR	0	2.738981281	0.403	0.049	0	Epithelial cell
MUC1	0	2.706017595	0.668	0.078	0	Epithelial cell
TM4SF4	0	2.656036851	0.59	0.042	0	Epithelial cell
MUC6	1.02E-108	2.613856486	0.201	0.097	3.30E-104	Epithelial cell
ADAMTS9	0	3.876694139	0.839	0.075	0	Endothelial cell
TCF4	0	3.588912269	0.947	0.176	0	Endothelial cell
ZNF385D	0	3.574882453	0.753	0.035	0	Endothelial cell
VWF	0	3.569547166	0.84	0.048	0	Endothelial cell
MCTP1	0	3.434462613	0.918	0.178	0	Endothelial cell
EMP1	0	3.410764652	0.93	0.218	0	Endothelial cell
ACKR1	0	3.358789683	0.625	0.027	0	Endothelial cell
CALCRL	0	3.307114464	0.874	0.037	0	Endothelial cell
SPARCL1	0	3.266484923	0.937	0.108	0	Endothelial cell
LDB2	0	3.241345019	0.892	0.065	0	Endothelial cell
TM4SF1	0	3.201365489	0.898	0.182	0	Endothelial cell
SLCO2A1	0	3.193838953	0.79	0.024	0	Endothelial cell
COL4A1	0	3.193774373	0.885	0.099	0	Endothelial cell
FLT1	0	3.156215309	0.86	0.064	0	Endothelial cell
SPRY1	0	3.147647502	0.835	0.16	0	Endothelial cell
CCL14	0	3.087868357	0.582	0.028	0	Endothelial cell
A2M	0	3.028232578	0.927	0.168	0	Endothelial cell
AQP1	0	3.018484053	0.814	0.046	0	Endothelial cell
WWTR1	0	2.917536038	0.896	0.134	0	Endothelial cell
RAPGEF4	0	2.913401189	0.824	0.023	0	Endothelial cell
EMCN	0	2.895862599	0.841	0.017	0	Endothelial cell
PLVAP	0	2.894971842	0.82	0.025	0	Endothelial cell
HSPG2	0	2.853125047	0.919	0.162	0	Endothelial cell
SELE	0	2.80142793	0.393	0.015	0	Endothelial cell
COL4A2	0	2.799920879	0.863	0.118	0	Endothelial cell
EPAS1	0	2.774955885	0.916	0.216	0	Endothelial cell
MAGI1	0	2.751448094	0.897	0.151	0	Endothelial cell
ADAMTS1	0	2.719997608	0.696	0.071	0	Endothelial cell
CAV1	0	2.691688397	0.805	0.079	0	Endothelial cell
ADGRL4	0	2.65389204	0.866	0.016	0	Endothelial cell
C1QA	0	3.89063701	0.487	0.027	0	Macrophage
C1QB	0	3.880380561	0.446	0.029	0	Macrophage
HLA-DRA	0	3.627233811	0.973	0.441	0	Macrophage
HLA-DPA1	0	3.567718571	0.896	0.415	0	Macrophage
HLA-DPB1	0	3.425154353	0.878	0.4	0	Macrophage
EREG	0	3.325925957	0.426	0.026	0	Macrophage
C1QC	0	3.162540417	0.41	0.014	0	Macrophage
HLA-DRB1	0	3.104971554	0.94	0.51	0	Macrophage
KYNU	0	3.074751727	0.664	0.047	0	Macrophage
IFI30	0	3.040926899	0.857	0.155	0	Macrophage
HLA-DQA1	0	2.998831628	0.696	0.194	0	Macrophage
APOE	0	2.965012004	0.256	0.058	0	Macrophage
IL1B	0	2.954961415	0.593	0.057	0	Macrophage
HLA-DQB1	0	2.868568236	0.81	0.275	0	Macrophage
HMOX1	0	2.77854088	0.507	0.055	0	Macrophage
CD74	0	2.685857086	0.971	0.653	0	Macrophage
AIF1	0	2.6758084	0.859	0.082	0	Macrophage
HLA-DRB5	0	2.594247645	0.43	0.106	0	Macrophage
FTL	0	2.542025121	0.988	0.915	0	Macrophage
APOC1	0	2.491219299	0.254	0.028	0	Macrophage
LYZ	0	2.443722604	0.848	0.294	0	Macrophage
CTSB	0	2.411376712	0.79	0.304	0	Macrophage
SLC16A10	0	2.401896793	0.383	0.044	0	Macrophage
SELENOP	1.69E-30	2.366839277	0.249	0.187	5.45E-26	Macrophage
CD14	0	2.366292444	0.6	0.054	0	Macrophage
TYROBP	0	2.337370546	0.909	0.161	0	Macrophage
MS4A6A	0	2.337314791	0.677	0.019	0	Macrophage
CCL3	0	2.269957589	0.467	0.089	0	Macrophage
FCER1G	0	2.210063471	0.835	0.12	0	Macrophage
CTSS	0	2.18672832	0.838	0.305	0	Macrophage
S100A8	0	5.135230924	0.883	0.075	0	Neutrophil
G0S2	0	5.015678811	0.88	0.088	0	Neutrophil
CXCL8	0	4.772440753	0.931	0.177	0	Neutrophil
NAMPT	0	4.345599152	0.994	0.606	0	Neutrophil
CCL3L1	0	4.109957587	0.437	0.136	0	Neutrophil
CSF3R	0	4.049324966	0.875	0.059	0	Neutrophil
S100A9	0	3.977599866	0.896	0.109	0	Neutrophil
FCGR3B	0	3.962493007	0.761	0.012	0	Neutrophil
LUCAT1	0	3.927239882	0.773	0.115	0	Neutrophil
AQP9	0	3.90473164	0.72	0.04	0	Neutrophil
PLAUR	0	3.875010777	0.867	0.28	0	Neutrophil
BCL2A1	0	3.794612326	0.864	0.184	0	Neutrophil
C15orf48	0	3.672037458	0.474	0.095	0	Neutrophil
SOD2	0	3.50694026	0.964	0.57	0	Neutrophil
LITAF	0	3.459225875	0.948	0.576	0	Neutrophil
IFITM2	0	3.45718279	0.922	0.596	0	Neutrophil
ALOX5AP	0	3.435853551	0.824	0.265	0	Neutrophil
CCL4L2	8.24E-283	3.428049127	0.473	0.217	2.66E-278	Neutrophil
PLEK	0	3.410378642	0.713	0.149	0	Neutrophil
BASP1	0	3.360817558	0.541	0.143	0	Neutrophil
LCP2	0	3.343282643	0.824	0.295	0	Neutrophil
DOCK4	0	3.295188665	0.816	0.259	0	Neutrophil
ACSL1	0	3.279946864	0.523	0.19	0	Neutrophil
IL1R2	0	3.241952307	0.556	0.047	0	Neutrophil
AZIN1-AS1	0	3.187173586	0.368	0.089	0	Neutrophil
RNF149	0	3.171311295	0.89	0.521	0	Neutrophil
LYN	0	3.154153081	0.803	0.238	0	Neutrophil
LIMK2	0	3.148992449	0.641	0.189	0	Neutrophil
LST1	0	3.147748078	0.734	0.104	0	Neutrophil
PHACTR1	0	3.116701357	0.716	0.186	0	Neutrophil
GNLY	0	3.858558691	0.485	0.042	0	NK cell
GZMB	0	3.293184761	0.726	0.073	0	NK cell
NKG7	0	2.906007893	0.831	0.149	0	NK cell
CCL5	0	2.576973484	0.911	0.262	0	NK cell
CXCL13	0	2.53384098	0.331	0.019	0	NK cell
GZMA	0	2.349490429	0.748	0.15	0	NK cell
KLRC1	0	2.065238897	0.492	0.014	0	NK cell
CCL4	0	2.004697283	0.75	0.221	0	NK cell
IFNG	0	1.939651141	0.576	0.087	0	NK cell
ITGAE	0	1.924947011	0.726	0.195	0	NK cell
TOX	0	1.909817993	0.779	0.212	0	NK cell
CD7	0	1.887301347	0.831	0.215	0	NK cell
ATP8B4	0	1.871117582	0.58	0.059	0	NK cell
PTPN22	0	1.849168772	0.89	0.304	0	NK cell
KLRD1	0	1.843229516	0.641	0.073	0	NK cell
GZMH	0	1.836999377	0.576	0.07	0	NK cell
LINC00299	0	1.824441467	0.577	0.041	0	NK cell
PRF1	0	1.820916776	0.675	0.076	0	NK cell
LINC01871	0	1.765495867	0.693	0.118	0	NK cell
CCL3	0	1.724953556	0.503	0.098	0	NK cell
XCL2	0	1.705719391	0.366	0.046	0	NK cell
AL136456.1	0	1.623449178	0.431	0.103	0	NK cell
RBPJ	0	1.603465772	0.806	0.437	0	NK cell
AC022126.1	0	1.581311541	0.397	0.033	0	NK cell
RGS1	0	1.576108977	0.726	0.282	0	NK cell
CD247	0	1.53503704	0.838	0.297	0	NK cell
HOPX	0	1.531538222	0.573	0.059	0	NK cell
KLRC2	0	1.506351637	0.424	0.013	0	NK cell
CTSW	0	1.485066923	0.538	0.063	0	NK cell
NCALD	0	1.470928648	0.637	0.142	0	NK cell
APOD	0	6.050551093	0.683	0.084	0	Fibroblast
LUM	0	4.888125883	0.834	0.058	0	Fibroblast
DCN	0	4.880080917	0.844	0.073	0	Fibroblast
PTGDS	0	4.771344077	0.55	0.061	0	Fibroblast
SFRP2	0	4.590442556	0.548	0.036	0	Fibroblast
COL1A1	0	4.51645072	0.783	0.105	0	Fibroblast
MGP	0	4.498968329	0.897	0.24	0	Fibroblast
COL3A1	0	4.41673769	0.791	0.08	0	Fibroblast
FBLN1	0	4.308291799	0.752	0.052	0	Fibroblast
COL1A2	0	4.259613198	0.806	0.092	0	Fibroblast
SERPINF1	0	3.75571127	0.725	0.05	0	Fibroblast
CCDC80	0	3.61298915	0.653	0.061	0	Fibroblast
C11orf96	0	3.520053302	0.783	0.147	0	Fibroblast
C1R	0	3.450216085	0.741	0.079	0	Fibroblast
CXCL14	0	3.437706518	0.308	0.015	0	Fibroblast
TIMP1	0	3.392610928	0.92	0.472	0	Fibroblast
CCN1	0	3.374978748	0.745	0.156	0	Fibroblast
CFD	0	3.334867069	0.466	0.078	0	Fibroblast
CCN2	0	3.292721212	0.725	0.14	0	Fibroblast
COL6A2	0	3.174406665	0.791	0.146	0	Fibroblast
C1S	0	3.119602326	0.723	0.105	0	Fibroblast
C7	0	3.097556568	0.578	0.069	0	Fibroblast
C3	0	3.091821258	0.57	0.119	0	Fibroblast
MMP2	0	2.98484368	0.7	0.08	0	Fibroblast
SPARC	0	2.967644036	0.799	0.204	0	Fibroblast
MFAP4	0	2.873302078	0.603	0.025	0	Fibroblast
PLA2G2A	0	2.870107235	0.242	0.014	0	Fibroblast
SFRP4	0	2.828696602	0.402	0.012	0	Fibroblast
COL6A3	0	2.768938434	0.64	0.021	0	Fibroblast
GEM	0	2.73346489	0.63	0.148	0	Fibroblast
IGLC2	0	6.849906924	0.478	0.119	0	B cell
IGKC	0	6.663837129	0.664	0.164	0	B cell
IGHA1	1.96E-291	5.717763579	0.451	0.124	6.31E-287	B cell
IGLC3	0	5.628362435	0.225	0.017	0	B cell
IGHG1	2.33E-280	5.072895022	0.249	0.039	7.52E-276	B cell
IGLC1	1.11E-244	4.402404884	0.173	0.022	3.56E-240	B cell
IGHG3	1.51E-205	4.185434362	0.192	0.031	4.88E-201	B cell
IGHA2	0	4.158321884	0.198	0.02	0	B cell
IGHM	0	3.911990821	0.496	0.013	0	B cell
JCHAIN	0	3.748060975	0.289	0.015	0	B cell
IGHG4	1.68E-77	3.68109683	0.152	0.044	5.42E-73	B cell
IGHG2	0	3.489411077	0.133	0.005	0	B cell
BANK1	0	3.214500103	0.775	0.023	0	B cell
AFF3	0	2.886735796	0.745	0.071	0	B cell
CD79A	0	2.8335787	0.836	0.009	0	B cell
GNG7	0	2.762703066	0.826	0.109	0	B cell
MS4A1	0	2.409367065	0.711	0.015	0	B cell
LY9	0	2.195457745	0.622	0.076	0	B cell
CD83	0	2.190611674	0.816	0.31	0	B cell
RALGPS2	0	2.170996119	0.716	0.126	0	B cell
ARHGAP24	0	2.115798024	0.732	0.147	0	B cell
EBF1	0	2.104186811	0.732	0.158	0	B cell
CD37	0	1.998503761	0.842	0.37	0	B cell
ADAM28	0	1.954540427	0.67	0.087	0	B cell
HLA-DQA1	0	1.881486171	0.814	0.209	0	B cell
ST6GAL1	0	1.841104143	0.744	0.25	0	B cell
CCR7	0	1.758775422	0.609	0.163	0	B cell
BACH2	1.10E-270	1.750314436	0.753	0.361	3.56E-266	B cell
LINC00926	0	1.728629684	0.567	0.012	0	B cell
AC120193.1	0	1.725218641	0.527	0.031	0	B cell
ITGB8	0	4.87318882	0.942	0.086	0	Nerve cell
NRXN1	0	4.768497028	0.915	0.01	0	Nerve cell
PPP2R2B	0	4.75644819	0.926	0.13	0	Nerve cell
CRYAB	0	4.457709408	0.855	0.057	0	Nerve cell
RUNX2	0	4.25276158	0.86	0.187	0	Nerve cell
NAV3	0	4.214723032	0.87	0.1	0	Nerve cell
FRMD5	0	4.161975142	0.892	0.058	0	Nerve cell
STARD13	0	4.138885722	0.926	0.161	0	Nerve cell
TENM3	0	4.063970191	0.886	0.038	0	Nerve cell
CDH19	0	4.019556371	0.883	0.011	0	Nerve cell
EHBP1	0	3.986690914	0.936	0.249	0	Nerve cell
COL8A1	0	3.968060897	0.825	0.031	0	Nerve cell
NRXN3	0	3.836876101	0.868	0.017	0	Nerve cell
GALNT17	0	3.702871986	0.834	0.014	0	Nerve cell
AL139383.1	0	3.593306627	0.808	0.064	0	Nerve cell
CCN3	0	3.532501682	0.714	0.031	0	Nerve cell
GAP43	0	3.439419315	0.811	0.012	0	Nerve cell
ZNF536	0	3.409694843	0.81	0.004	0	Nerve cell
GPM6B	0	3.397168883	0.844	0.057	0	Nerve cell
DST	0	3.318283481	0.935	0.224	0	Nerve cell
SESN3	0	3.30528539	0.837	0.231	0	Nerve cell
ANK3	0	3.212172313	0.933	0.296	0	Nerve cell
CADM2	0	3.060011361	0.696	0.011	0	Nerve cell
SEMA3B	0	3.028846172	0.855	0.075	0	Nerve cell
ADGRB3	0	3.016330199	0.695	0.042	0	Nerve cell
NRP2	0	3.015978753	0.849	0.132	0	Nerve cell
SORCS1	0	2.974886773	0.741	0.018	0	Nerve cell
FN1	0	2.931418143	0.809	0.126	0	Nerve cell
SLC22A23	0	2.928784324	0.673	0.081	0	Nerve cell
ADAMTS9-AS2	0	2.906337313	0.819	0.127	0	Nerve cell
TPSB2	0	6.419787202	0.921	0.029	0	Mast cell
TPSAB1	0	6.085651326	0.914	0.016	0	Mast cell
CPA3	0	4.863626313	0.924	0.005	0	Mast cell
SLC24A3	0	4.092509583	0.938	0.042	0	Mast cell
HPGDS	0	3.848203148	0.878	0.017	0	Mast cell
HPGD	0	2.936250164	0.677	0.067	0	Mast cell
CTSG	0	2.828868123	0.352	0.002	0	Mast cell
MS4A2	0	2.815825714	0.774	0.002	0	Mast cell
HDC	0	2.759363017	0.736	0.006	0	Mast cell
GATA2	0	2.732908987	0.73	0.041	0	Mast cell
FER	0	2.720937805	0.877	0.287	0	Mast cell
AREG	1.36E-214	2.67197275	0.675	0.272	4.38E-210	Mast cell
LTC4S	0	2.607220423	0.655	0.094	0	Mast cell
KIT	0	2.564888405	0.724	0.018	0	Mast cell
ACSL4	0	2.512046759	0.82	0.283	0	Mast cell
SLC18A2	0	2.466939247	0.703	0.024	0	Mast cell
ABCC4	0	2.280137013	0.687	0.126	0	Mast cell
IL1RL1	0	2.221268846	0.713	0.008	0	Mast cell
VWA5A	0	2.217745349	0.649	0.036	0	Mast cell
ADAM12	0	2.193481109	0.645	0.03	0	Mast cell
CSF1	0	2.172964007	0.576	0.113	0	Mast cell
RAB27B	0	2.121452093	0.605	0.037	0	Mast cell
RHEX	0	2.089479665	0.659	0.028	0	Mast cell
ENPP3	0	2.06776375	0.438	0.007	0	Mast cell
IL18R1	0	2.060176401	0.691	0.124	0	Mast cell
ALOX5	0	2.037115764	0.699	0.106	0	Mast cell
RGS13	0	2.016974258	0.672	0.042	0	Mast cell
ANXA1	1.33E-245	1.962156692	0.917	0.598	4.29E-241	Mast cell
CADPS	0	1.939507966	0.531	0.021	0	Mast cell
ADGRE2	0	1.926089903	0.667	0.079	0	Mast cell
TAGLN	0	5.475161646	0.9	0.125	0	Tissue stem cell
ACTA2	0	4.971979812	0.867	0.101	0	Tissue stem cell
C11orf96	0	4.518464263	0.912	0.161	0	Tissue stem cell
MYL9	0	4.290246475	0.917	0.125	0	Tissue stem cell
TPM2	0	4.187163834	0.906	0.13	0	Tissue stem cell
MYH11	0	4.084321297	0.741	0.058	0	Tissue stem cell
ADIRF	0	3.903358852	0.902	0.153	0	Tissue stem cell
RGS5	0	3.569655911	0.499	0.031	0	Tissue stem cell
MUSTN1	0	3.482426649	0.659	0.011	0	Tissue stem cell
CRISPLD2	0	3.457782966	0.805	0.076	0	Tissue stem cell
PRKG1	0	3.409746635	0.844	0.117	0	Tissue stem cell
CALD1	0	3.409170838	0.942	0.227	0	Tissue stem cell
SOD3	0	3.226520727	0.808	0.043	0	Tissue stem cell
IGFBP7	0	3.124695849	0.96	0.296	0	Tissue stem cell
DSTN	0	3.113584738	0.927	0.441	0	Tissue stem cell
SPARCL1	0	2.970323941	0.908	0.194	0	Tissue stem cell
TPM1	0	2.95328782	0.851	0.22	0	Tissue stem cell
CSRP2	0	2.871080931	0.802	0.064	0	Tissue stem cell
PPP1R14A	0	2.85268515	0.791	0.036	0	Tissue stem cell
PDK4	0	2.828109884	0.666	0.122	0	Tissue stem cell
MFGE8	0	2.66823482	0.817	0.088	0	Tissue stem cell
PDE3A	0	2.660932195	0.759	0.042	0	Tissue stem cell
NDUFA4L2	0	2.627323903	0.539	0.01	0	Tissue stem cell
NOTCH3	0	2.576897674	0.814	0.025	0	Tissue stem cell
PDGFRB	0	2.565128239	0.763	0.038	0	Tissue stem cell
SOX5	0	2.550861642	0.788	0.111	0	Tissue stem cell
CEBPD	0	2.53665668	0.842	0.251	0	Tissue stem cell
RERGL	0	2.526515505	0.52	0.007	0	Tissue stem cell
SORBS2	0	2.496744624	0.624	0.106	0	Tissue stem cell
CSRP1	0	2.469935441	0.747	0.19	0	Tissue stem cell

*dCCA*, Distal cholangiocarcinoma.

**Supplementary Table 2:** eCCA Classifier Containing 174 Genes

Gene	Cluster
ORM1	Metabolic
FGA	Metabolic
ALB	Metabolic
HP	Metabolic
APOA2	Metabolic
AGT	Metabolic
ITIH3	Metabolic
ITIH2	Metabolic
SERPINA6	Metabolic
ITIH4	Metabolic
FGG	Metabolic
PCK1	Metabolic
VTN	Metabolic
SLC25A47	Metabolic
FGB	Metabolic
AZGP1	Metabolic
SERPINA1	Metabolic
SERPINC1	Metabolic
SLC13A5	Metabolic
ITIH1	Metabolic
KNG1	Metabolic
CYP27A1	Metabolic
MGST1	Metabolic
CYP2B6	Metabolic
PBLD	Metabolic
APOC2	Metabolic
APOC3	Metabolic
AHSG	Metabolic
C9	Metabolic
ALDH4A1	Metabolic
CFB	Metabolic
APOB	Metabolic
ADH1B	Metabolic
TF	Metabolic
CYP3A4	Metabolic
APOA1	Metabolic
CYP2E1	Metabolic
VNN1	Metabolic
SEPP1	Metabolic
SERPINA3	Metabolic
APCS	Metabolic
EPHX1	Metabolic
CBS	Metabolic
RBP4	Metabolic
UGT2B4	Metabolic
CP	Metabolic
CYP1A2	Metabolic
CPS1	Metabolic
TTC39C	Metabolic
CYP3A5	Metabolic
RPL28	Proliferation
RPL37A	Proliferation
STAG3L2	Proliferation
WAC	Proliferation
CLIC1	Proliferation
RPS3	Proliferation
POM121C	Proliferation
PIK3C2A	Proliferation
STAG3L3	Proliferation
YWHAB	Proliferation
RPL11	Proliferation
EEF2	Proliferation
PTPN2	Proliferation
RPL36A	Proliferation
SERP1	Proliferation
EFTUD2	Proliferation
CNOT7	Proliferation
STK25	Proliferation
UXT	Proliferation
RPL41	Proliferation
RPS29	Proliferation
TMBIM6	Proliferation
6-Mar	Proliferation
HDGF	Proliferation
PAPOLA	Proliferation
GNB2L1	Proliferation
SYNCRIP	Proliferation
ATP5L	Proliferation
MRPL42	Proliferation
FAU	Proliferation
KHSRP	Proliferation
SPINT1	Proliferation
RPL4	Proliferation
RBM17	Proliferation
RPL14	Proliferation
SMARCE1	Proliferation
MORF4L2	Proliferation
MRPS24	Proliferation
UBE2N	Proliferation
UBA52	Proliferation
OST4	Proliferation
TAPBP	Proliferation
H2AFY	Proliferation
RPS15A	Proliferation
SLC25A6	Proliferation
UPF3A	Proliferation
RPL5	Proliferation
SNW1	Proliferation
TUBB2C	Proliferation
MRFAP1	Proliferation
POSTN	Mesenchymal
THBS2	Mesenchymal
SYTL4	Mesenchymal
NRP2	Mesenchymal
INHBA	Mesenchymal
NEURL	Mesenchymal
COL1A1	Mesenchymal
COL12A1	Mesenchymal
OLFML2B	Mesenchymal
SPARC	Mesenchymal
COL10A1	Mesenchymal
CDH11	Mesenchymal
TAGLN	Mesenchymal
BHLHE40	Mesenchymal
ITGA11	Mesenchymal
VCAN	Mesenchymal
MYL9	Mesenchymal
CALD1	Mesenchymal
STON1	Mesenchymal
GREM1	Mesenchymal
LGALS1	Mesenchymal
VIM	Mesenchymal
TGFBI	Mesenchymal
COMP	Mesenchymal
HTRA3	Mesenchymal
FAM127A	Mesenchymal
COL11A1	Mesenchymal
CCDC80	Mesenchymal
EHD2	Mesenchymal
OSBPL5	Mesenchymal
COL5A1	Mesenchymal
PALLD	Mesenchymal
WIPF1	Mesenchymal
IVNS1ABP	Mesenchymal
SULF1	Mesenchymal
TIMP2	Mesenchymal
SH3PXD2B	Mesenchymal
FSTL1	Mesenchymal
C5AR1	Mesenchymal
COL6A1	Mesenchymal
PTK7	Mesenchymal
PLOD2	Mesenchymal
DNAJB5	Mesenchymal
CDH13	Mesenchymal
CRISPLD2	Mesenchymal
PLK3	Mesenchymal
COL1A2	Mesenchymal
ROR2	Mesenchymal
SERPINE1	Mesenchymal
LSAMP	Mesenchymal
IGH	Immune
IGHA1	Immune
PIK3IP1	Immune
CORO1A	Immune
IGJ	Immune
ATP2A3	Immune
ARHGDIB	Immune
IGHM	Immune
PTGDS	Immune
HIST1H2AE	Immune
SMAP2	Immune
PAPSS1	Immune
ITGAX	Immune
CD4	Immune
IL23A	Immune
ARHGAP9	Immune
CCDC69	Immune
HIST1H2BD	Immune
CCR7	Immune
CYR61	Immune
NR4A1	Immune
HIST1H4E	Immune
TSC22D3	Immune
TCF7	Immune

*eCCA*, Extrahepatic cholangiocarcinoma.

## References

[1] Banales J.M., Marin J.G., Lamarca A., Rodrigues P.M., Khan S.A., Roberts R., Vincenzo C., Guido C., Andersen J.B., Chiara B., Calvisi D.F., Perugorria M.J., Fabris L., Boulter L., Macias R., Gaudio E., Alvaro D., Gradilone S.A., Strazzabosco M., Marzioni M., Coulouarn C., Fouassier L., Raggi C., Invernizzi P., Mertens J.C., Moncsek A., Rizvi S., Heimbach J., Koerkamp B.C., Bruix J., Forner A., Bridgewater J., Valle J.W., Gores G.J. (2020). Cholangiocarcinoma 2020: the next horizon in mechanisms and management. Nat Rev Gastroenterol Hepatol.

[2] Moeini A., Haber P.K., Sia D. (2021). Cell of origin in biliary tract cancers and clinical implications. JHEP Rep.

[3] Valle J.W., Kelley R.K., Nervi B., Oh D.Y., Zhu A.X. (2021). Biliary tract cancer. Lancet.

[4] Zhou W., Qian L., Rong Y., Zhou Q., Shan J., Li P., Shi L., Liu H., Sun X. (2020). Prognostic factors and patterns of recurrence after curative resection for patients with distal cholangiocarcinoma. Radiother Oncol.

[5] Tanguy L.C., Turrini O., Bergeat D., Truant S., Darnis B., Delpero J.R., Mabrut J.Y., Regenet N., Sulpice L. (2018). Multicentre study of the impact of factors that may affect long-term survival following pancreaticoduodenectomy for distal cholangiocarcinoma. HPB (Oxford).

[6] Komaya K., Ebata T., Shirai K., Ohira S., Morofuji N., Akutagawa A., Yamaguchi R., Nagino M., Nagoya Surgical Oncology Group (2017). Recurrence after resection with curative intent for distal cholangiocarcinoma. Br J Surg.

[7] Wardell C.P., Fujita M., Yamada T., Simbolo M., Fassan M., Karlic R., Polak P., Kim J., Hatanaka Y., Maejima K., Lawlor R.T., Nakanishi Y., Mitsuhashi T., Fujimoto A., Furuta M., Ruzzenente A., Conci S., Oosawa A., Sasaki-Oku A., Nakano K., Tanaka H., Yamamoto Y., Michiaki K., Kawakami Y., Aikata H., Ueno M., Hayami S., Gotoh K., Ariizumi S., Yamamoto M., Yamaue H., Chayama K., Miyano S., Getz G., Scarpa A., Hirano S., Nakamura T., Nakagawa H. (2018). Genomic characterization of biliary tract cancers identifies driver genes and predisposing mutations. J Hepatol.

[8] Suva M.L., Tirosh I. (2019). Single-cell RNA sequencing in cancer: lessons learned and emerging challenges. Mol Cell.

[9] Zhang P., Yang M., Zhang Y., Xiao S., Lai X., Tan A., Du S., Li S. (2019). Dissecting the single-cell transcriptome network underlying gastric premalignant lesions and early gastric cancer. Cell Rep.

[10] Zhang Q., He Y., Luo N., Patel S.J., Han Y., Gao R., Modak M., Carotta S., Haslinger C., Kind D., Peet G.W., Zhong G., Lu S., Zhu W., Mao Y., Xiao M., Bergmann M., Hu X., Kerkar S.P., Vogt A.B., Pflanz S., Liu K., Peng J., Ren X., Zhang Z. (2019). Landscape and dynamics of single immune cells in hepatocellular carcinoma. Cell.

[11] Zhang M., Yang H., Wan L., Wang Z., Wang H., Ge C., Liu Y., Hao Y., Zhang D., Shi G., Gong Y., Ni Y., Wang C., Zhang Y., Xi J., Wang S., Shi L., Zhang L., Yue W., Pei X., Liu B., Yan X. (2020). Single-cell transcriptomic architecture and intercellular crosstalk of human intrahepatic cholangiocarcinoma. J Hepatol.

[12] Macosko E.Z., Basu A., Satija R., Nemesh J., Shekhar K., Goldman M., Tirosh I., Bialas A.R., Kamitaki N., Martersteck E.M., Trombetta J.J., Weitz D.A., Sanes J.R., Shalek A.K., Regev A., McCarroll S.A. (2015). Highly parallel genome-wide expression profiling of individual cells using nanoliter droplets. Cell.

[13] Korsunsky I., Millard N., Fan J., Slowikowski K., Zhang F., Wei K., Baglaenko Y., Brenner M., Loh P.R., Raychaudhuri S. (2019). Fast, sensitive and accurate integration of single-cell data with Harmony. Nat Methods.

[14] Dann E., Henderson N.C., Teichmann S.A., Morgan M.D., Marioni J.C. (2022). Differential abundance testing on single-cell data using k-nearest neighbor graphs. Nat Biotechnol.

[15] Lun A.T.L., Richard A.C., Marioni J.C. (2017). Testing for differential abundance in mass cytometry data. Nat Methods.

[16] Yang Y.G., Leonard M., Luo Z.H., Yeo S., Bick G., Hao M.G., Cai C., Charif M., Wang J., Guan J., Lower E.E., Zhang X. (2021). Functional cooperation between co-amplified genes promotes aggressive phenotypes of HER2-positive breast cancer. Cell Rep.

[17] Yu C., Luo D., Yu J., Zhang M., Zheng X., Xu G., Wang J., Wang H., Xu Y., Jiang K., Xu J., Ma X., Jing J., Shi H. (2022). Genome-wide CRISPR-cas9 knockout screening identifies GRB7 as a driver for MEK inhibitor resistance in KRAS mutant colon cancer. Oncogene.

[18] Naba A., Clauser K.R., Lamar J.M., Carr S.A., Hynes R.O. (2014). Extracellular matrix signatures of human mammary carcinoma identify novel metastasis promoters. Elife.

[19] Noll E.M., Eisen C., Stenzinger A., Espinet E., Muchenhuber A., Klein C., Vogel V., Klaus B., Nadler W., Rosli C., Lutz C., Kulke M., Engelhardt J., Zickgraf F.M., Espinosa O., Schlesner M., Jiang X., Schneider A.K., Neuhaus P., Bahra M., Sinn B.V., Eils R., Giese N.A., Hackert T., Strobel O., Werner J., Buchler M.W., Weichert W., Trumpp A., Sprick M.R. (2016). CYP3A5 mediates basal and acquired therapy resistance in different subtypes of pancreatic ductal adenocarcinoma. Nat Med.

[20] Shi J., Wang X., Ding G., Dong Z., Han J., Guan Z., Ma L., Zheng Y., Zhang L., Yu G., Wang X., Ding Z., Ke A., Yang H., Wang L., Ai L., Cao Y., Zhou J., Fan J., Liu X., Gao Q. (2021). Exploring prognostic indicators in the pathological images of hepatocellular carcinoma based on deep learning. Gut.

[21] Xiao Y., Rabien A., Buschow R., Amtislavskiy V., Busch J., Kilic E., Villegas S.L., Timmermann B., Schutte M., Mielke T., Yaspo M.L., Jung K., Meierhofer D. (2020). Endocytosis-mediated replenishment of amino acids favors cancer cell proliferation and survival in chromophobe renal cell carcinoma. Cancer Res.

[22] Li X., Bechara R., Zhao J., McGeachy M.J., Gaffen S.L. (2019). Interleukin 17 receptor-based signaling and implications for disease. Nat Immunol.

[23] Yu H., Pardoll D., Jove R. (2009). STATs in cancer inflammation and immunity: a leading role for STAT3. Nat Rev Cancer.

[24] Hunter J.E., Leslie J., Perkins N.D. (2016). C-Rel and its many roles in cancer: an old story with new twists. Br J Cancer.

[25] Chen G., Zhou H., Liu B., Wang Y., Zhao J., Giancotti F.G., Long J. (2020). A heterotrimeric SMARCB1-SMARCC2 subcomplex is required for the assembly and tumor suppression function of the BAF chromatin-remodeling complex. Cell Discov.

[26] Li L., Ameri A.H., Wang S., Jansson K.H., Casey O.M., Yang Q., Beshiri M.L., Fang L., Lake R.G., Agarwal S., Alilin A.N., Xu W., Ying J., Kelly K. (2019). EGR1 regulates angiogenic and osteoclastogenic factors in prostate cancer and promotes metastasis. Oncogene.

[27] Ireland A.S., Micinski A.M., Kastner D.W., Guo B., Wait S.J., Spainhower K.B., Conley C.C., Chen O.S., Guthrie M.R., Soltero D., Qiao Y., Huang X., Tarapcsak S., Devarakonda S., Chalishazar M.D., Gertz J., Moser J.C., Marth G., Puri S., Witt B.L., Spike B.T., Oliver T.G. (2020). MYC drives temporal evolution of small cell lung cancer subtypes by reprogramming neuroendocrine fate. Cancer Cell.

[28] Street K., Risso D., Fletcher R.B., Das D., Ngai J., Yosef N., Purdom E., Dudoit S. (2018). Slingshot: cell lineage and pseudotime inference for single-cell transcriptomics. BMC Genomics.

[29] Li T., Shen K., Li J., Leung S., Zhu T., Shi Y. (2021). Glomerular endothelial cells are the coordinator in the development of diabetic nephropathy. Front Med.

[30] Montal R., Sia D., Montironi C., Leow W.Q., Fabro R.E., Pinyol R., Martin M.T., Bassaganyas L., Moeini A., Peix J., Cabellos L., Maeda M., Martin C.V., Tabrizian P., Carunchio L.R., Castellano G., Sempoux C., Minguez B., Pawlik T.M., Labgaa I., Roberts L.R., Sole M., Fiel M.I., Thung S., Fuster J., Roayaie S., Villanueva A., Schwartz M., Llovert J.M. (2020). Molecular classification and therapeutic targets in extrahepatic cholangiocarcinoma. J Hepatol.

[31] Rizvi S., Gores G.J. (2013). Pathogenesis, diagnosis, and management of cholangiocarcinoma. Gastroenterology.

[32] Tiwari R., Manzar N., Bhatia V., Yadav A., Nengroo M.A., Datta D., Carskadon S., Gupta N., Sigouros M., Khani F., Poutanen M., Zoubeidi A., Beltran H., Palanisamy N., Ateeq B. (2020). Androgen deprivation upregulates SPINK1 expression and potentiates cellular plasticity in prostate cancer. Nat Commun.

[33] Huang K., Xie W., Wang S., Li Q., Wei X., Chen B., Hua Y., Li S., Peng B., Shen S. (2021). High SPINK1 expression predicts poor prognosis and promotes cell proliferation and metastasis of hepatocellular carcinoma. J Invest Surg.

[34] Morse C., Tabib T., Sembrat J., Buschur K.L., Bittar H.T., Valenzi E., Jiang Y., Kass D.J., Gibson K., Chen W., Mora A., Benos P.V., Rojas M., Lafyatis R. (2019). Proliferating SPP1/MERTK-expressing macrophages in idiopathic pulmonary fibrosis. Eur Respir J.

[35] Taupin D., Podolsky D.K. (2003). Trefoil factors: initiators of mucosal healing. Nat Rev Mol Cell Biol.

[36] Dhar D.K., Wang T., Tabara H., Tonomoto Y., Maruyama R., Tachibana M., Kubota H., Nagasue N. (2005). Expression of trefoil factor family members correlates with patient prognosis and neoangiogenesis. Clin Cancer Res.

[37] Reames B.N., Rocha F.G. (2021). Early recurrence following resection of distal cholangiocarcinoma: a new tool for the toolbox. Ann Surg Oncol.

[38] Zhang M., Hu S., Min M., Ni Y., Lu Z., Sun X., Wu J., Liu B., Ying X., Liu Y. (2021). Dissecting transcriptional heterogeneity in primary gastric adenocarcinoma by single cell RNA sequencing. Gut.

[39] Zhang Y., Narayanan S.P., Mannan R., Raskind G., Wang X., Vats P., Su F., Hosseini N., Cao X., Sinha C.K., Ellison S.J., Giordano T.J., Morgan T.M., Pitchiaya S., Alva A., Mehra R., Cieslik M., Dhanasekaran S.M., Chinnaiyan A.M. (2021). Single-cell analyses of renal cell cancers reveal insights into tumor microenvironment, cell of origin, and therapy response. Proc Natl Acad Sci U S A.

[40] Wang R., Dang M., Harada K., Han G., Wang F., Pizzi M.P., Zhao M., Tatlonghari G., Zhang S., Hao D., Lu Y., Zhao S., Badgwell B.D., Murphy M.B., Shanbhag N., Estrella J.S., Chowdhuri S.R., Abdelhakeem A.F., Wang Y., Peng G., Hanash S., Calin G.A., Song X., Chu Y., Zhang J., Li M., Chen K., Lazar A.J., Futreal A., Song S., Ajani J.A., Wang L. (2021). Single-cell dissection of intratumoral heterogeneity and lineage diversity in metastatic gastric adenocarcinoma. Nat Med.

[41] Mitsopoulos C., Micco P.D., Fernandez E.V., Dolciami D., Holt E., Mica I.L., Coker E.A., Tym J.E., Campbell J., Che K.H., Ozer B., Kannas C., Antolin A.A., Workman P., Lazikani B.A. (2021). canSAR: update to the cancer translational research and drug discovery knowledgebase. Nucleic Acids Res.

[42] Finn O.J. (2008). Molecular origins of cancer: cancer immunology. N Engl J Med.

[43] Galon J., Fridman W.H., Pages F. (2007). The adaptive immunologic microenvironment in colorectal cancer: a novel perspective. Cancer Res.

[44] Puram S.V., Tirosh I., Parikh A.S., Patel A.P., Yizhak K., Gillespie S., Rodman C., Luo C.L., Mroz E.A., Emerick K.S., Deschler D.G., Varvares M.A., Mylvaganam R., Rosen O.R., Rocco J.W., Faquin W.C., Lin D.T., Regev A., Bernstein B.E. (2017). Single-cell transcriptomic analysis of primary and metastatic tumor ecosystems in head and neck cancer. Cell.

[45] Gattinoni L., Klebanoff C.A., Palmer D.C., Wrzesinski C., Kerstann K., Yu Z.Y., Finkelstein S.E., Theoret M.R., Rosenberg S.A., Restifo N.P. (2005). Acquisition of full effector function in vitro paradoxically impairs the in vivo antitumor efficacy of adoptively transferred CD8+ T cells. J Clin Invest.

[46] Jain A.S., Drygin D., Streiner N., Chua P., Pierre F., O’Brien S.E., Bliesath J., Omori M., Huser N., Ho C., Proffitt C., Schwaebe M.K., Ryckman D.M., Rice W.G., Anderes K. (2010). CX-4945, an orally bioavailable selective inhibitor of protein kinase CK2, inhibits prosurvival and angiogenic signaling and exhibits antitumor efficacy. Cancer Res.

[47] On W.C., Nairismagi M.L., Ong C.K., Lin W.K., Dima S., Pairojkul C., Lim K.H., McPherson J.R., Cutcutache I., Heng H.L., Ooi L., Chung A., Chow P., Cheow P.C., Lee S.Y., Choo S.P., Tan I.B., Duda D., Nastase A., Myint S.S., Wong B.H., Gan A., Rajasegaran V., Young C.C., Nagarajan S., Jusakul A., Zhang S., Vohra P., Yu W., Huang D., Sithithaworn P., Yongvanit P., Wongkham S., Khuntikeo N., Bhudhisawasdi V., Popescu I., Rozen S.G., Tan P., Teh B.T. (2013). Exome sequencing identifies distinct mutational patterns in liver fluke-related and non-infection-related bile duct cancers. Nat Genet.

[48] Jusakul A., Cutcutache I., Yong C.H., Lim J.Q., Huang M.N., Padmanabhan N., Nellore V., Kongpetch S., Ng A.W.T., Ng L.M., Choo S.P., Myint S.S., Thanan R., Nagarajan S., Lim W.K., Ng C.C.Y., Boot A., Liu M., Ong C.K., Rajasegaran V., Lie S., Lim A.S., Lim T.H., Tan J., Loh J.L., McPherson J.R., Khuntikeo N., Bhudhisawasdi V., Yongvanit P., Wongkham S., Totoki Y., Nakamura H., Arai Y., Yamasaki S., Chow P.K., Chung A.Y., Peng L.L., Lim K.H., Dima S., Duda D.G., Popescu I., Broet P., Hsieh S.Y., Yu M.C., Scarpa A., Lai J., Luo D.X., Carvalho A.L., Vettore A.L., Rhee H., Park Y.N., Alexandrov L.B., Gordan R., Rozen S.G., Shibata T., Pairojkul C., Teh B.T., Tan P. (2017). Whole-genome and epigenomic landscapes of etiologically distinct subtypes of cholangiocarcinoma. Cancer Discov.

[49] Nakamura H., Arai Y., Totoki Y., Shirota T., Elzawahry A., Kato M., Hama N., Hosoda F., Urushidate T., Ohashi S., Hiraoka N., Ojima H., Shimada K., Okusaka T., Kosuge T., Miyagawa S., Shibata T. (2015). Genomic spectra of biliary tract cancer. Nat Genet.

[50] Satija R., Farrell J.A., Gennert D., Schier A.F., Regev A. (2015). Spatial reconstruction of single-cell gene expression data. Nat Biotechnol.

[51] Tirosh I., Izar B., Prakadan S.M., Wadsworth M.H., Treacy D., Trombetta J.J., Rotem A., Rodman C., Lian C., Murphy G., Sichani M.F., Regester K.D., Lin J.R., Cohen O., Shah P., Lu D., Genshaft A.S., Hughes T.K., Ziegler C.G., Kazer S.W., Gaillard A., Kolb K.E., Villani A.C., Johannessen C.M., Andreev A.Y., Alhen E.M., Bertagnolli M., Sorger P.K., Sullivan R.J., Flaherty K.T., Frederick D.T., Valbuena J.J., Yoon C.H., Rosen O.R., Shalek A.K., Regev A., Garraway L.A. (2016). Dissecting the multicellular ecosystem of metastatic melanoma by single-cell RNA-seq. Science.

[52] Abdelaal T., Michielsen L., Cats D., Hoogduin D., Mei H., Reinders M.J., Mahfouz A. (2019). A comparison of automatic cell identification methods for single-cell RNA sequencing data. Genome Biol.

[53] Chen W.J., Pan X.W., Chu J., Xu D., Chen J.X., Chen W.J., Wang L.H., Cui X.G. (2021). Study of cellular heterogeneity and differential dynamics of autophagy in human embryonic kidney development by single-cell RNA sequencing. Cancer Cell Int.

[54] Trapnell C., Cacchiarelli D., Grimsby J., Pokharel P., Li S., Morse M., Lennon N.J., Livak K.J., Mikkelsen T.S., Rinn J.L. (2014). The dynamics and regulators of cell fate decisions are revealed by pseudotemporal ordering of single cells. Nat Biotechnol.

