# Correction

**DOI:** 10.1016/j.jcmgh.2026.101903

**Published:** 2026-10-31

Li H, Qu L, Yang Y, et al. Single-cell Transcriptomic Architecture Unraveling the Complexity of Tumor Heterogeneity in Distal Cholangiocarcinoma. Cell Mol Gastroenterol Hepatol 2022;13:1592-1609.e9.

In the above article, the authors wish to clarify the availability of the scRNA-seq data in this study. The scRNA-seq data have been deposited at the Genome Sequence Archive at the National Genomics Data Center (Beijing, China) under accession code GSA-Human: HRA002645 and are publicly accessible at https://ngdc.cncb.ac.cn/gsa-human/.

